# The Immune-Senescent Switch in Oral Carcinogenesis: From Premalignancy to Malignant Transformation—Does Periodontitis Tip the Balance? A Hypothesis-Driven Review

**DOI:** 10.3390/diagnostics16213466

**Published:** 2026-10-23

**Authors:** Shujing Lyu, Eva González-Angulo

**Affiliations:** School of Medicine and Stomatology, University of Valencia, 46010 Valencia, Spain

**Keywords:** immune checkpoint inhibitors, immunosenescence, inflammaging, malignant transformation, oral potentially malignant disorders, oral squamous cell carcinoma, periodontitis, senescence-associated secretory phenotype, senolytics, translational therapeutics

## Abstract

Oral squamous cell carcinoma (OSCC) accounts for approximately 90% of oral malignancies, and most cases arise from oral potentially malignant disorders (OPMDs) against a background of chronic inflammation. Periodontitis, a highly prevalent chronic inflammatory disease, is increasingly implicated as a risk factor for OSCC, yet the mechanistic link between these conditions remains unclear. This review proposes that immunosenescence—the age-related decline in immune function—may provide this link. We synthesise evidence indicating that periodontitis promotes immune ageing in oral and systemic immune compartments, encompassing T-cell exhaustion, the accumulation of senescent immune cells, and a pro-inflammatory senescence-associated secretory phenotype (SASP); together, these changes progressively weaken local immunosurveillance. On this basis, we introduce the “immune-senescent switch” as a conceptual framework—explicitly distinguished from established evidence—in which senescence is hypothesised to initially act as a tumour-suppressive barrier in early OPMDs but to be converted into a tumour-promoting force by chronic, periodontitis-driven immunosenescence. We critically evaluate the supporting evidence, including the single-arm phase 2 COIS-01 trial (*n* = 24), in which senolytics combined with anti-PD-1 therapy achieved a 33.3% major pathological response rate in resectable head and neck squamous cell carcinoma, while emphasising the preliminary, hypothesis-generating nature of these data. Finally, we discuss emerging senotherapeutic strategies, translational gaps, and future research priorities.

## 1. Introduction

Oral squamous cell carcinoma (OSCC) accounts for around 90% of all oral malignancies and remains a significant global health concern, with 5-year survival rates at approximately 50% [1]. Despite advances in surgery, radiotherapy, and immunotherapy, late detection and therapeutic resistance continue to compromise outcomes [2]. Most OSCCs develop from oral potentially malignant disorders (OPMDs), including leukoplakia, erythroplakia, and oral submucous fibrosis [3], with malignant transformation occurring most frequently in lesions showing histological dysplasia. It is thought that a persistently pro-inflammatory oral microenvironment drives this transition [4].

Among the contributors to this inflammatory milieu, periodontitis is a particularly compelling candidate. Afflicting around 50% of the global population, it is one of the most prevalent chronic diseases worldwide. Epidemiological evidence increasingly implicates periodontitis as an independent risk factor for OSCC. A systematic review and meta-analysis of 18 studies involving 31,504 participants found an odds ratio (OR) of 3.35 (95% confidence interval (CI): 2.63–4.26; *p* < 0.001) for oral cancer in individuals with periodontal disease. The adjusted OR remained significant at 2.32 (95% CI: 1.71–3.15; *p* < 0.001) after controlling for confounders [5]. In a hospital-based case–control study of 266 incident head and neck squamous cell carcinoma cases and 207 controls, patients with periodontitis were more likely than those without periodontitis to have poorly differentiated oral cavity squamous cell carcinoma (32.8% versus 11.5%; *p* = 0.038) [6]. Notably, however, a recent two-sample Mendelian randomisation study did not support a causal relationship between periodontitis and oral cancer [7], illustrating that genetic causal inference and clinicopathological association address different levels of evidence. Nevertheless, residual confounding factors, particularly tobacco use, alcohol consumption, and socioeconomic status, may partly explain this association. Some studies have reported attenuation or loss of significance after comprehensive adjustment for these factors, suggesting that the independent effect of periodontitis may be more modest than unadjusted estimates imply [8,9].

The oncogenic influence of periodontitis appears to extend beyond the oral cavity. Epidemiological studies have linked periodontal disease to various systemic malignancies, suggesting shared pathways involving chronic inflammation and immunosenescence [9,10].

In parallel with these observations, the global population is ageing, and advanced age is the most significant risk factor for both periodontitis and OSCC. At the heart of ageing biology lies inflammaging—a chronic, low-grade, systemic pro-inflammatory state sustained by the interplay of cellular, immunological, and metabolic senescence [4]. These interconnected processes disrupt tissue homeostasis, heighten disease susceptibility, and drive the functional decline of multiple organ systems. The pathogenesis of periodontitis, oral mucosal disorders and their malignant transformation is increasingly understood in the context of inflammaging and systemic degeneration. Age-related shifts in oral microbial composition, including increased colonisation by periodontal pathogens such as *Porphyromonas gingivalis*, have been documented in older adults (65–80 years) and correlate with elevated serum interleukin-6 (IL-6) and C-reactive protein (CRP) levels compared with younger individuals [11]. Experimental gingivitis in healthy older adults induces systemic endotoxemia, with plasma endotoxin levels rising from 50 to 201 pg/mL, accompanied by increases in soluble CD14. Encouragingly, periodontal treatment reduces serum CRP and IL-6 within two months [11].

Notably, periodontitis has emerged as an immune-related adverse event associated with immune checkpoint inhibitors, suggesting that the periodontal inflammatory milieu directly intersects with cancer immunotherapy pathways [12]. Furthermore, the subgingival microbial community in patients with both periodontitis and OSCC exhibits distinct compositional characteristics, and heterogeneity in the tumour tissue T-cell receptor β (TCRβ) complementarity-determining region 3 (CDR3) repertoire indicates localised immune dysregulation [13].

In preparing this synthesis, we have sought to distinguish clearly between established evidence and the conceptual model we propose. Throughout this review, statements are anchored to their level of support—epidemiological association, preclinical experimentation, or clinical trial data—and the “immune-senescent switch” is explicitly presented as a hypothesis-generating framework that integrates, rather than restates, the existing literature. Where evidence is conflicting or preliminary, this is stated explicitly, and the strength of the evidence supporting each mechanistic link is critically appraised (Section 4.7).

Literature search and selection. As a hypothesis-driven narrative review, this article does not follow a systematic review protocol. The literature was identified through structured searches of PubMed/MEDLINE, Scopus, and Web of Science from database inception to September 2026, using combinations of the terms “periodontitis”, “periodontal disease”, “oral squamous cell carcinoma”, “oral potentially malignant disorder”, “immunosenescence”, “cellular senescence”, “senescence-associated secretory phenotype”, “senolytic”, “immune checkpoint”, “PD-1/PD-L1”, “cGAS-STING”, and “oral microbiome”. Peer-reviewed original studies, systematic reviews and meta-analyses, and clinical trials published in English were eligible; priority was given to human and in vivo evidence, landmark mechanistic studies, and the most recent high-quality sources, and the reference lists of key articles were screened for additional relevant studies. The strength of the evidence supporting each central claim is appraised as described in Section 4.7.

## 2. Immunosenescence: Hallmarks and Mechanisms

Immunosenescence refers to the progressive deterioration of the immune system with ageing. It is characterised by a decline in adaptive immunity, chronic low-grade inflammation, and impaired immune surveillance. This process substantially increases the risk of developing cancer; approximately 60% of new cancer cases and 70% of cancer-related deaths occur in individuals aged 65 years or older [14]. A recent comprehensive review identified eleven molecular hallmarks of immunosenescence, including genomic instability, telomere attrition, epigenetic dysregulation, mitochondrial dysfunction, and chronic inflammation, that collectively drive immune cell dysfunction and systemic immunosuppression [15]. Ageing remodels the tumour microenvironment by recruiting immunosuppressive cells, amplifying the senescence-associated secretory phenotype (SASP), and altering metabolism. This fosters therapy resistance and a poor prognosis in elderly patients [16,17].

In the oral cavity, these age-related immune alterations have significant consequences. The oral mucosa is a unique immunological interface that is constantly challenged by mechanical, microbial, and chemical stimuli. Immunosenescence within this compartment may therefore impair local immune surveillance disproportionately, creating permissive conditions for OPMD progression.

### 2.1. Adaptive Immune Senescence: The T-Cell Compartment

The T-cell compartment is particularly susceptible to immunosenescence. After puberty, thymic involution progressively depletes the pool of naïve T cells, leading to the development of senescent CD4+ and CD8+ T cells. To sustain T-cell homeostasis, the remaining naïve T cells undergo extensive homeostatic proliferation, eventually reaching their replicative limit [18]. Senescent T cells characteristically express programmed cell death protein 1 (PD-1) and CD153, exhibit diminished proliferative capacity and resistance to apoptosis, and display an enhanced SASP. Cellular senescence should be distinguished from T-cell exhaustion: senescence denotes a durable cell-cycle arrest programme characterised by senescence-associated β-galactosidase (SA-β-gal) activity and p16^INK4A^/p21 upregulation, whereas exhaustion denotes a dysfunctional state driven by persistent antigenic stimulation and characterised by sustained co-expression of inhibitory receptors (PD-1, CTLA-4, TIM-3, LAG-3) with progressive loss of effector cytokine production. The two states may coexist and partially overlap, but they are not interchangeable; throughout this review, we indicate which state each cited study actually assessed.

In a cancer setting, senescent T cells demonstrate impaired effector functions, reduced cytolytic activity, and defective tumour-cell recognition [16]. Tumour-infiltrating senescent CD8+ T cells exhibit functional alterations that may blunt their responsiveness to immune checkpoint inhibitors. Single-cell multi-omics profiling of head and neck squamous cell carcinoma (HNSCC) patients has revealed that non-responsive tumours bear prominent immunosenescence signatures, including reduced CCR7+ CD4+ naïve T cells and CD27+ memory B cells [19]. Ageing oral mucosa during infection also shows increased accumulation of CD4+ Foxp3+ regulatory T cells associated with immune dysfunction [20].

### 2.2. Innate Immune Senescence: Macrophages and Nk Cells

The impact of ageing on innate immune cells, particularly macrophages and natural killer (NK) cells, has received less attention than T-cell senescence. However, emerging evidence suggests that aged macrophages exhibit altered polarisation, reduced phagocytic capacity, and increased secretion of pro-inflammatory cytokines. In oral cancer, oxidative stress drives tumour microenvironment remodelling by inducing macrophage senescence. Senescent macrophages promote OSCC invasion via glutamine metabolic reprogramming, establishing a direct mechanistic link between innate immune cell senescence and tumour progression.

NK cells, which are key effectors of anti-tumour innate immunity, also decline in function with age. Aged NK cells exhibit reduced cytotoxicity, impaired cytokine production, and altered receptor expression, which compromises their ability to recognise and eliminate malignant cells.

### 2.3. The Senescence-Associated Secretory Phenotype

Perhaps the most significant feature of senescent cells, whether immune or non-immune, is the acquisition of the SASP. These cells secrete a variety of pro-inflammatory cytokines, chemokines, growth factors, and matrix-remodelling enzymes, including IL-6, IL-8, tumour necrosis factor-α (TNF-α), and multiple matrix metalloproteinases (MMPs) [21]. The SASP is not static, but rather a dynamic programme that varies depending on the cell type and context, with both beneficial and deleterious effects. Its molecular regulation involves complex signalling networks, including the nuclear factor-κB (NF-κB), p38 mitogen-activated protein kinase (MAPK), and mechanistic target of rapamycin (mTOR) pathways, offering both therapeutic opportunities and translational challenges [22].

In the short term, the SASP reinforces cell-cycle arrest, recruits immune cells to clear senescent cells and supports tissue repair. However, when senescent cells persist—as occurs with ageing and chronic inflammation—the SASP becomes chronically sustained, driving maladaptive inflammation, immune dysfunction, vascular perturbation, and extracellular matrix remodelling. This chronic, SASP-driven inflammation is central to inflammaging and substantially contributes to age-related diseases, including cancer [22]. Continuous SASP expression disrupts immune homeostasis in the oral microenvironment [23]. In head and neck cancers, persistent DNA damage response signalling sustains the SASP. This, in turn, modulates the tumour microenvironment, facilitates epithelial–mesenchymal transition, promotes cancer stem cell-like properties, and impairs the efficacy of radiotherapy, chemotherapy and immune checkpoint inhibitors. The senescence-associated cytokines IL-6 and IL-8 establish a self- and cross-reinforcing inflammatory milieu that amplifies tumourigenic potential [24].

Quantitative analyses support the diagnostic potential of SASP factors in oral cancer, with IL-6 and C-X-C motif chemokine ligand 1 (CXCL1) showing robust performance in distinguishing malignant from normal tissues; their prognostic and treatment-predictive value remains to be established (see Section 6.2 for a detailed discussion).

## 3. Periodontitis as a Driver of Immunosenescence

Periodontitis and immunosenescence engage in a reciprocal, self-reinforcing loop. While ageing predisposes individuals to periodontitis through immunosenescence, periodontitis in turn accelerates immunosenescence via sustained inflammatory signalling [25].

### 3.1. Periodontitis Induces Systemic Immune Senescence

Experimental evidence shows that severe periodontal inflammation promotes the senescence of helper T cells. In a murine ligature-induced periodontitis model, splenic CD4+ T cells from mice with periodontitis showed increased senescence-associated β-galactosidase (SA-β-gal) positivity, elevated SASP cytokine levels, and a reduced S-phase fraction—hallmarks of accelerated senescence [25,26]. RNA sequencing analysis revealed numerous differentially expressed senescence-related genes in helper T cells from mice with periodontitis [26]. Importantly, the adoptive transfer of these senescent CD4+ T cells into nude mice exacerbated collagen antibody-induced arthritis, demonstrating that periodontitis-induced senescent T cells can enter the circulation and promote systemic inflammatory disease [26]. Complementary causal evidence comes from p16-3MR reporter mice, in which the selective clearance of p16^INK4A^-positive senescent cells during ligature-induced periodontitis alleviated local inflammation and mitigated alveolar bone loss, establishing a causal contribution of senescent cells to periodontal pathology [27]. Microbiota-induced exosomes from dendritic cells have also been identified as potent drivers of alveolar bone loss and immune senescence. This suggests that immune senescence, compounded by advanced age and accelerated by oral dysbiosis and its SASP-containing exosomes, plays a pivotal role in experimental periodontitis [28].

Taken together, these findings reveal a cellular mechanism that links periodontitis to systemic immunosenescence. Therefore, periodontitis emerges not merely as a consequence of age-related immune decline, but as an active accelerator of immune ageing.

### 3.2. Periodontitis and the Pd-1/Pd-L1 Immune Checkpoint Axis

Periodontitis has a significant impact on the PD-1/programmed death-ligand 1 (PD-L1) immune checkpoint pathway, which is a key regulator of T-cell exhaustion and immune surveillance. We note that checkpoint engagement reflects T-cell exhaustion rather than cellular senescence per se (see Section 2.1); unless otherwise stated, the studies cited below assessed checkpoint expression and T-cell dysfunction, not senescence markers. Chronic periodontal inflammation reprograms innate and adaptive immune responses through shared signalling cascades, including NF-κB, signal transducer and activator of transcription 3 (STAT3), and PD-1/PD-L1 [29].

Importantly, periodontitis promotes tumour growth and immune evasion by increasing PD-1/PD-L1 expression within the tumour microenvironment. In mouse oral cancer xenograft models, periodontitis was found to significantly increase tumour volume and weight. Compared with the control group, the periodontitis group showed elevated proportions of tumour-associated macrophages (TAMs), regulatory T cells, PD-L1+ TAMs, and PD-1+ CD8+ T cells, alongside reduced CD8+ T cells [29]. Immunofluorescence confirmed an increase in PD-L1+ TAMs and PD-1+ CD8+ T cells, but a decrease in interferon-γ-positive (IFN-γ+) CD8+ T cells, in both xenografts and clinical OSCC specimens from patients with periodontitis. In vitro, lipopolysaccharide (LPS)-stimulated OSCC cells induced PD-L1 expression in macrophages more potently than unstimulated cells did. Anti-PD-1 therapy significantly reduced the tumour-promoting effect of periodontitis. Furthermore, gingipains from *P. gingivalis* promote immune evasion by modulating the alternative splicing of PD-L1 in macrophages, selectively upregulating a coding isoform that binds PD-1 on T cells more effectively [30]. PD-L1 upregulation in OSCC correlates with immune escape and poor prognosis, implying that periodontitis-induced checkpoint dysregulation creates a permissive niche for OSCC progression [29].

At a molecular level, *P. gingivalis* cell wall components, including peptidoglycans delivered via outer membrane vesicles, induce PD-L1 expression in human oral carcinoma cells via a receptor-interacting protein 2 (RIP2)-dependent mechanism. This offers a direct pathway of pathogen–host interaction linking periodontal infection to immune checkpoint upregulation in the oral mucosa [31].

### 3.3. Periodontitis-Induced Myeloid Reprogramming

In addition to its effects on T cells, periodontitis alters the differentiation of myeloid cells, resulting in an immunosuppressive phenotype. It drives OSCC progression and fosters an immunosuppressive tumour microenvironment that is rich in myeloid-derived suppressor cells, tumour-associated macrophages, and regulatory T cells. High-risk OSCC tumours exhibit CD8+ T-cell depletion, M0 macrophage infiltration, and the upregulation of immunosuppressive markers. This myeloid skewing is particularly relevant to immunosenescence, given that aged macrophages exhibit altered polarisation and function, and the accumulation of immunosuppressive myeloid cells is characteristic of the tumour microenvironment in older individuals.

### 3.4. The Oral Microbiome–Immunosenescence Axis

Periodontitis-associated microbial dysbiosis further exacerbates immunosenescence. *Fusobacterium nucleatum*, an anaerobe that accumulates in oral tumour tissues, has attracted particular attention as it is thought to facilitate both tumourigenesis and chemoresistance. Its ability to adhere to epithelial cells, invade tissues, subvert local immunity, and polarise macrophages towards an immunosuppressive phenotype establishes it as a pivotal agent in the connection between periodontal inflammation and tumour progression [32]. A systematic review and meta-analysis of 16 case–control studies (1608 participants) confirmed significant enrichment of *F. nucleatum* in OSCC patients across multiple sample types. At the phylum level, the OSCC group showed reduced levels of Actinobacteria (standardised mean difference (SMD) = −0.85) and increased levels of Fusobacteria (SMD = 0.61). At the genus level, *Fusobacterium*, *Capnocytophaga*, *Peptostreptococcus*, *Alloprevotella*, *Parvimonas*, and *Dialister* were significantly enriched, while *Streptococcus*, *Haemophilus*, *Rothia*, *Veillonella*, *Actinomyces*, and *Leptotrichia* were depleted [33]. These microbial signatures offer a quantitative framework for understanding the oral dysbiosis–immunosenescence–carcinogenesis axis.

Periodontal pathogens such as *P. gingivalis* and *F. nucleatum* drive oral carcinogenesis by modulating molecular, genetic, and epigenetic pathways, including p53, Cyclin D1, p16^INK4A^, DNA methylation, histone modifications, and microRNA regulation [34]. The interplay between the oral microbiome and the immune system in ageing populations may generate conditions that permit carcinogenesis and tumour progression. Age-associated immunosenescence, chronic low-grade inflammation and dysbiosis across the oral and gastrointestinal microbiomes are increasingly recognised as interconnected drivers of cancer risk in older adults [35].

### 3.5. Porphyromonas Gingivalis Outer Membrane Vesicles Suppress Innate Immune Surveillance via Cgas-Sting Inhibition

A 2025 study revealed that outer membrane vesicles (OMVs) of the bacterium *Porphyromonas gingivalis* promote oral tumour formation by suppressing the innate immune signalling of the cyclic GMP-AMP synthase–stimulator of interferon genes (cGAS-STING) [36]. *P. gingivalis* was significantly enriched in HNSCC tissues, including OSCC, with increased abundance in both saliva and tumour specimens correlating with an advanced clinical stage. The uptake of *P. gingivalis* OMVs by OSCC cells was confirmed in both in vitro and in vivo models.

OMVs are critical virulence factors produced by Gram-negative bacteria, including *P. gingivalis*, that facilitate intercellular communication, immune modulation, and the delivery of pathogenic cargo to host cells [37,38]. The composition and functions of bacterial membrane vesicles have been extensively characterised, revealing their involvement in processes such as biofilm formation, immune evasion, and horizontal gene transfer [38].

Mechanistically, *P. gingivalis* OMVs disrupted both interferon regulatory factor 3 (IRF3)–STING and IRF3–TANK-binding kinase 1 (TBK1) interactions, thereby blocking downstream signalling. In both immunodeficient (BALB/c-nu) and immunocompetent (C57BL/6) mouse models, *P. gingivalis* OMVs suppressed cGAS-STING signalling, thereby impairing IFN-β production and limiting the recruitment of natural killer cells and dendritic cells to the tumour microenvironment. Treatment with herring testis DNA partially restored pIRF3 translocation and induced IFN-β production in vivo. These findings establish *P. gingivalis* OMVs as potent blockers of intrinsic immune surveillance. cGAS, a cytosolic DNA sensor that activates the type I interferon pathway, is a cornerstone of innate anti-tumour immunity [39]. The STING polymer structure reveals the mechanisms of activation, hyperactivation and inhibition, providing a structural basis for understanding how *P. gingivalis* OMVs disrupt this pathway [40]. This discovery provides a direct mechanistic link between periodontal pathogens and oral cancer immune evasion, complementing the PD-1/PD-L1 axis and expanding our understanding of how periodontitis undermines immunosurveillance. Furthermore, the release of mitochondrial DNA by senescent tumour cells enhances polymorphonuclear myeloid-derived suppressor cell (PMN-MDSC)-driven immunosuppression via the cGAS-STING pathway, indicating that senescence and periodontal infection converge on this innate immune axis to promote tumour progression [41]. These two sets of observations are not necessarily contradictory: OMV-mediated suppression acts on innate sensing in epithelial and immune cells early and locally, whereas mitochondrial DNA released by senescent tumour cells activates STING signalling within PMN-MDSCs in established tumours; the net effect of cGAS-STING modulation is therefore cell-type-, duration-, and context-dependent. Moreover, altered cGAS-STING signalling does not by itself establish immunosenescence; we cite this pathway as a point of convergence between infection and senescence rather than as evidence of senescence per se. Accordingly, STING agonists are listed in Table 1 as exploratory, preclinical agents rather than as near-term therapeutic candidates.

## 4. The Immune-Senescent Switch in Oral Carcinogenesis

### 4.1. The Proposed Conceptual Model

**Figure 1:**
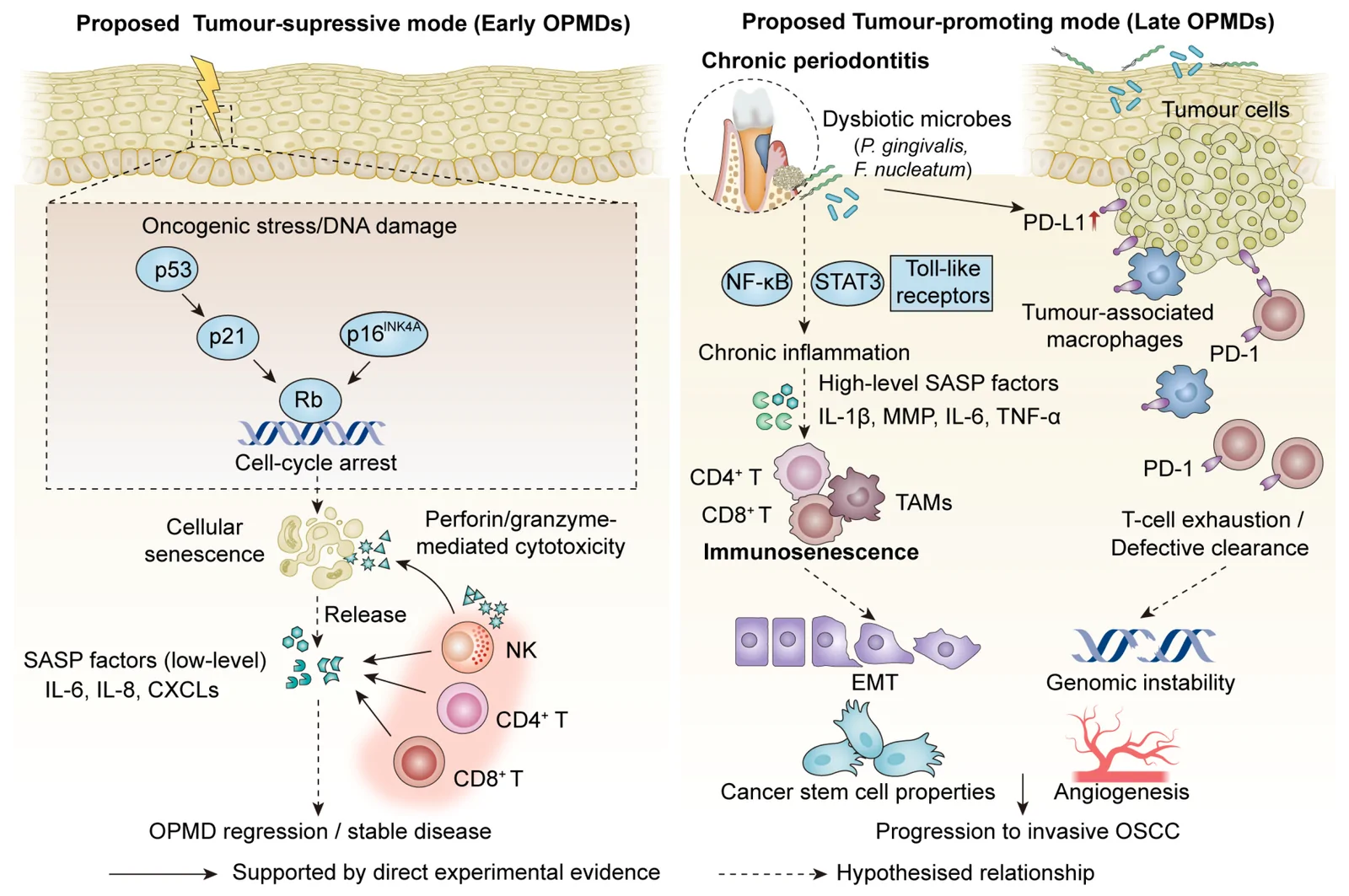
The immune-senescent switch in oral carcinogenesis. This schematic illustrates the proposed dual and context-dependent roles of cellular senescence during progression from oral potentially malignant disorders to oral squamous cell carcinoma. In the proposed tumour-suppressive mode (early OPMDs), epithelial cells undergoing senescence enforce stable cell-cycle arrest via p53/p21 and p16^INK4A^/Rb pathways. These senescent cells secrete low levels of SASP factors that chemoattract NK cells and CD8+ T lymphocytes, which eliminate senescent and premalignant cells through cytotoxicity. In the proposed tumour-promoting mode (late OPMDs), chronic periodontitis is hypothesised to accelerate immunosenescence, marked by accumulation of senescent T cells, macrophages, and exhausted immune subsets. These immunosenescent cells acquire a chronic, high-level SASP with excessive secretion of pro-inflammatory cytokines, matrix-remodelling enzymes, and growth factors. Concurrently, periodontitis upregulates PD-L1 on tumour-associated macrophages and tumour cells while promoting PD-1 on CD8+ T cells, driving T-cell exhaustion and impairing immune-mediated clearance. The sustained SASP milieu, coupled with defective immune clearance, promotes epithelial–mesenchymal transition, genomic instability, cancer stem cell-like properties, and angiogenesis, ultimately facilitating OPMD progression to invasive OSCC. The central arrow indicates the hypothesised tipping point at which periodontitis-driven immunosenescence would flip the switch from tumour suppression to tumour promotion. Solid arrows denote relationships supported by direct experimental evidence; dashed arrows denote hypothesised relationships proposed in this review. p16^INK4A^ (the *CDKN2A* gene product) is depicted as a single protein.

The evidence reviewed above suggests a conceptual framework in which immunosenescence plays a key role in determining whether senescence promotes or constrains oral carcinogenesis (Figure 1). This framework is particularly relevant in OPMDs, where the outcome of malignant transformation depends on the balance between immune surveillance and immune evasion.

It must be emphasised from the outset that the “immune-senescent switch” is a conceptual model proposed in this review, not an established mechanism. Each of its components is supported by evidence of a different type and strength, and the model as a whole has not been demonstrated directly. Based on the available evidence, one can conceptualise an “immune-senescent switch”: a threshold at which the net effect of senescence in the oral premalignant microenvironment shifts from tumour suppression to tumour promotion. The available data suggest that three interdependent parameters govern this transition: (1) the burden of senescent cells (epithelial and immune), which appears to dictate the magnitude of SASP output; (2) the composition of SASP, which seems to shift from a profile predominantly driven by chemokines that recruit immune cells to a profile predominantly driven by cytokines that promote inflammation and persistent senescence; and (3) the functional integrity of immune effector cells, which determines whether senescent cells are cleared efficiently or allowed to accumulate. Periodontitis appears to accelerate the crossing of this threshold by increasing the burden of senescent cells (via chronic inflammatory DNA damage), remodelling the composition of the SASP (via NF-κB/STAT3 hyperactivation), and impairing immune clearance (via PD-1/PD-L1 upregulation and cGAS-STING suppression). The following sections examine the evidence for each of these propositions in turn.

To make this model experimentally tractable, we specify how such a switch could be operationalised and measured. We propose that pre- and post-switch states can be distinguished along the three governing parameters: (i) senescent-cell burden, measurable as the density of SA-β-gal-positive and p16^INK4A^/p21-positive cells (epithelial and immune) in lesional and adjacent tissue; (ii) SASP activity, measurable as a panel of SASP effectors (e.g., IL-6, IL-8, CXCL1, TNF-α, and MMPs) in tissue, saliva, or serum; and (iii) immune-effector integrity, measurable as the CD8+/regulatory T-cell ratio, IFN-γ production, the frequency of CCR7+ naïve T cells, and the clearance rate of senescent cells. We do not claim that the transition is necessarily abrupt or discrete at the single-cell level: epithelial-protective and stromal- or immune-associated pro-tumourigenic senescence may coexist within the same lesion, and the “switch” is best understood as the point at which the net, population-level effect of senescence crosses from tumour-suppressive to tumour-promoting. This operational definition distinguishes the model from gradual, unstructured deterioration and renders it falsifiable.

### 4.2. Senescence as a Tumour-Suppressive Barrier in Early Opmds

In its protective mode, cellular senescence acts as a potent tumour-suppressive mechanism. It suppresses malignancy in premalignant epithelia by enforcing stable cell-cycle arrest in cells at risk of neoplastic transformation [42]. In early OPMDs, senescent cells recruit immune cells, such as NK cells and CD8+ T cells, through SASP-mediated chemokine signalling, thereby promoting the immune-mediated clearance of damaged or potentially malignant cells [22]. Throughout this review, we use “early OPMDs” to denote lesions without dysplasia or with low-grade (mild-to-moderate) epithelial dysplasia, and “late OPMDs” to denote lesions with high-grade (severe) dysplasia or carcinoma in situ. OPMDs are a heterogeneous group of disorders with variable, non-obligatory progression to malignancy [3]; our model therefore applies most directly to dysplasia-bearing lesions and should not be taken to imply that all OPMDs follow a uniform senescence-switch trajectory.

This immune-mediated clearance is a critical component of cancer immunosurveillance. Senescent cells display surface markers that make them recognisable to the immune system, and their SASP facilitates the recruitment and activation of immune effectors. In the oral cavity, senescence contributes to maintaining tissue integrity, supporting wound repair and suppressing malignant transformation [42]. Keratinocyte senescence acts as a barrier to tumour progression in early premalignancy. However, this barrier appears to be lost in late premalignancy, resulting in genetically unstable OSCC. We argue that the transition from senescence-mediated suppression to senescence-driven promotion is a critical inflection point in oral carcinogenesis that is modulated by immunosenescence.

Preclinical studies using the 4-nitroquinoline-1-oxide (4NQO) mouse model of oral carcinogenesis have provided direct experimental evidence of the immunopreventive potential of restoring T-cell function at the premalignant stage. Systemic PD-1 blockade in p53+/− mice exposed to 4NQO significantly reduced the incidence of high-grade lesions (severe dysplasia and carcinoma) by 43.3%. This was accompanied by increased infiltration of CD8+ and CD4+ T cells, signal transducer and activator of transcription 1 (STAT1) activation, and granzyme B production in oral lesions [47]. Similarly, PD-1 blockade has been shown to effectively inhibit the malignant transformation of the oral mucosa in mice exposed to carcinogens, thereby restoring T-cell cytotoxicity and slowing the progression of premalignant lesions [48]. These findings demonstrate that modulating immune checkpoints at the premalignant stage can reactivate immunosurveillance and prevent malignant transformation. We emphasise, however, that these 4NQO studies documented reversible T-cell exhaustion and the effects of checkpoint blockade, not cellular senescence; they therefore support the immune-dysfunction component of our model but do not, by themselves, establish an immune-senescent switch.

Recent evidence further clarifies the temporal dynamics of T-cell dysfunction during oral carcinogenesis. Using the 4NQO model, investigators demonstrated that features of T-cell exhaustion, including the upregulation of PD-1, cytotoxic T-lymphocyte-associated protein 4 (CTLA-4), T-cell immunoglobulin and mucin-domain-containing protein 3 (TIM-3), and lymphocyte-activation gene 3 (LAG-3), emerge in the early premalignant phase (as early as 4–8 weeks of carcinogen exposure) and precede histological evidence of dysplasia. Notably, the secretion of cytokines (IL-2, TNF-α and IFN-γ) by both CD4+ and CD8+ T cells declined progressively with disease progression. PD-1 blockade initiated at the early premalignant phase was found to effectively reverse T-cell exhaustion and prevent carcinogenic progression, whereas delayed intervention was less effective [46]. These data suggest that immune dysfunction emerges early in oral carcinogenesis—consistent with, though not proof of, an early engagement of the proposed switch—and that therapeutic intervention must precede the development of chronic T-cell exhaustion to be effective.

### 4.3. Immunosenescence Flips the Switch: From Suppression to Promotion

When immunosenescence takes hold, whether due to ageing, chronic periodontitis, or both, the tumour-suppressive functions of senescence are progressively eroded. This transition is driven by four interconnected mechanisms.

Firstly, senescent immune cells themselves become sources of chronic SASP. Senescent CD4+ CD153+ T cells, SA-β-gal+ macrophages, and dysfunctional NK cells secrete pro-inflammatory cytokines (such as IL-6, IL-8, TNF-α, and MMPs) that sustain inflammaging within the oral microenvironment. These senescent immune cells have been reported to accumulate in chronically inflamed gingival tissues and are hypothesised to extend into adjacent OPMD tissues, creating localised inflammatory foci that promote epithelial–mesenchymal transition, genomic instability, and stemness acquisition in premalignant cells [49,50]. The spatial distribution of senescent immune cells between periodontal and OPMD tissues has not, however, been mapped directly (see Section 6.1). Several anatomical routes could link the two compartments—local diffusion of soluble SASP mediators, salivary transport of pathogens and their products, systemic circulating inflammation, and trafficking of senescent immune cells from periodontal sites. These routes carry differing levels of supporting evidence, which future spatial studies should seek to distinguish.

Secondly, immunosenescence impairs the clearance of senescent cells. As we age and experience chronic inflammation, our immune system becomes less efficient at recognising and eliminating these cells. The resulting accumulation of these cells amplifies SASP-driven inflammation, creating a vicious cycle of senescence accumulation and immune dysfunction.

Thirdly, senescent stromal cells within the tumour microenvironment actively promote OSCC progression. Through the secretion of inflammatory factors via the SASP, senescent cells facilitate the proliferation and malignant transformation of dysplastic cells.

Fourthly, immunosenescence-driven immune checkpoint dysregulation promotes immune evasion. As mentioned previously, periodontitis increases PD-1/PD-L1 expression. In OPMDs, this upregulation of the checkpoint may prematurely exhaust T cells that would otherwise eliminate emerging malignant clones, thereby permitting the outgrowth of transformed cells.

A p16-dependent increase in PD-L1 stability regulates the immunosurveillance of senescent cells, driving their resistance to anti-PD-1-mediated clearance and thereby highlighting a direct mechanistic link between senescence and resistance to checkpoint inhibitors [44]. Blocking PD-L1–PD-1 signalling improves senescence surveillance and ameliorates ageing phenotypes, suggesting that checkpoint modulation may restore immune clearance of senescent cells [45].

At the microbial level, intratumoural *Fusobacterium nucleatum* has emerged as a key modulator of the immune-senescent switch. In OSCC, enrichment of *F. nucleatum* correlates significantly with immune-related gene expression profiles and identifies a patient subgroup with distinct clinical characteristics. *F. nucleatum*-positive tumours exhibit altered immune cell infiltration, displaying an inverse correlation between bacterial load and CD163+ M2 macrophage density. This suggests that *F. nucleatum* modulates the myeloid compartment within the tumour microenvironment [51]. Furthermore, *P. gingivalis* and *F. nucleatum* act synergistically to promote OSCC progression. Co-infection of oral cancer cells with both pathogens significantly enhances proliferation and migration compared with single infections. Transcriptomic analyses reveal accelerated cell cycle progression and amplified pro-inflammatory signalling [52]. These findings demonstrate that periodontal pathogens do not act in isolation, but rather cooperatively drive the transition from tumour suppression to tumour promotion. Beyond the oral cavity, *F. nucleatum* drives the enrichment of cancer-associated fibroblasts and immune exclusion in oesophageal squamous cell carcinoma through NF-κB-mediated inflammatory signalling. This provides a paradigm for understanding how oral bacteria remodel the tumour stroma to facilitate immune evasion [53].

### 4.4. The Opmd–Periodontitis–Immunosenescence Triangle

**Figure 2:**
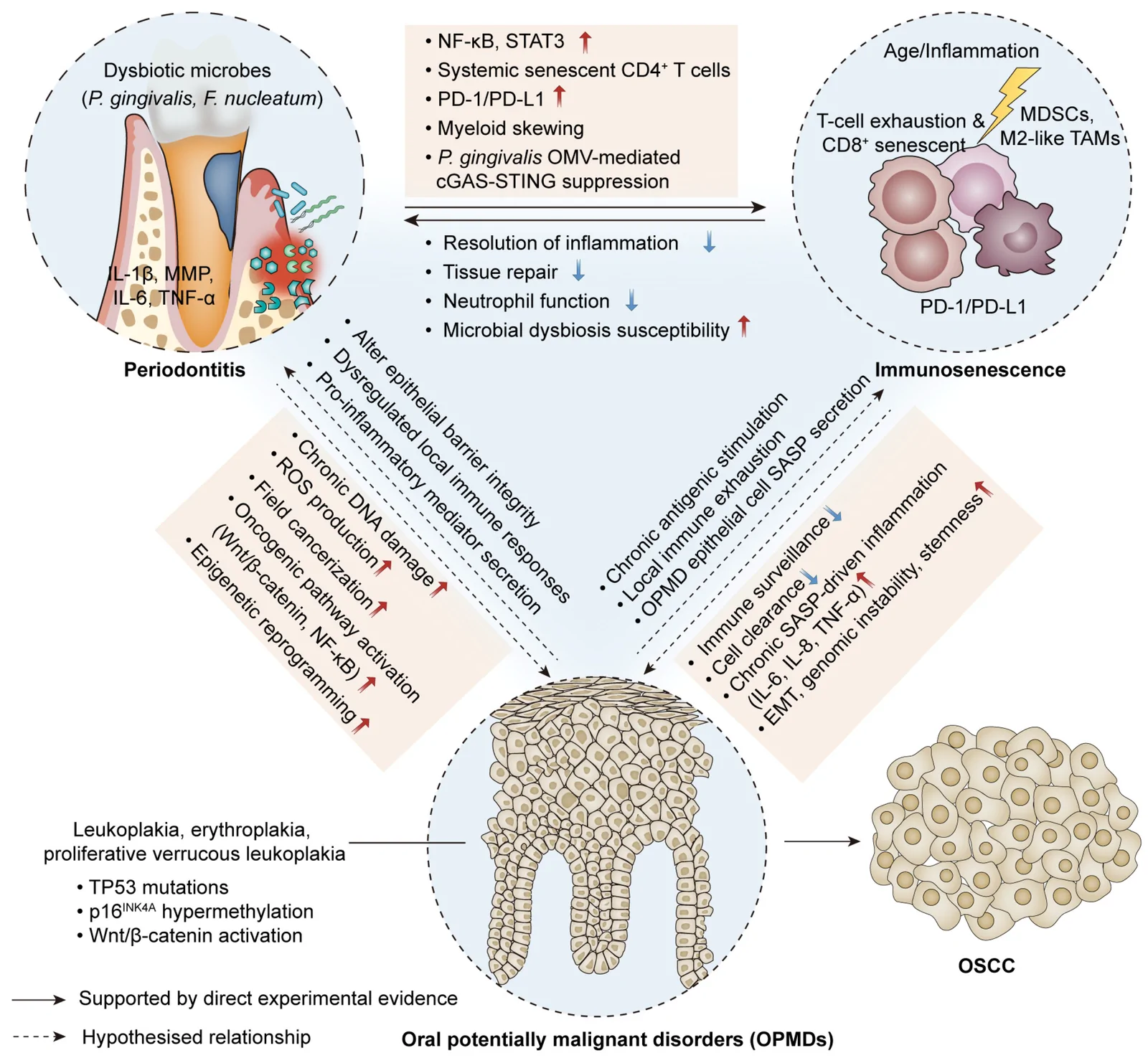
The OPMD–periodontitis–immunosenescence triangular pathogenic model. This conceptual framework illustrates three interconnected pathogenic vertices—periodontitis, immunosenescence, and OPMD—and their bidirectional, feed-forward relationships. Periodontitis (Vertex 1) is a chronic inflammatory disease driven by microbial dysbiosis, with a sustained host inflammatory response and local production of pro-inflammatory mediators. It serves as the initial inflammatory driver perpetuating a pro-tumourigenic oral microenvironment. Immunosenescence (Vertex 2) represents age-related and inflammation-accelerated progressive immune decline, encompassing T-cell exhaustion, accumulation of senescent macrophages, myeloid skewing towards immunosuppressive subsets, PD-1/PD-L1 checkpoint upregulation, and chronic maladaptive SASP. It is proposed as the mechanistic bridge connecting chronic inflammation to impaired tumour immune surveillance. OPMD (Vertex 3) encompasses oral potentially malignant disorders that may harbour dysplastic epithelial cells with accumulated genetic and epigenetic alterations. These lesions exist in precarious equilibrium between immune-mediated elimination and malignant outgrowth. Bidirectional arrows connect the vertices: periodontitis drives immunosenescence through sustained inflammatory signalling, systemic dissemination of senescent CD4+ T cells, PD-1/PD-L1 upregulation, myeloid skewing, and *P. gingivalis* OMV-mediated cGAS-STING suppression. Immunosenescence impairs immune surveillance in OPMDs, reducing clearance of senescent and dysplastic cells, while OPMDs further drive immunosenescence through chronic antigenic stimulation and SASP secretion. Periodontitis directly contributes to OPMD progression through chronic inflammation-induced DNA damage and oncogenic pathway activation, while OPMDs reciprocally exacerbate adjacent periodontal inflammation. At the centre lies chronic SASP-driven inflammation and immune dysregulation—the functional core of this feed-forward loop. Solid arrows denote established or experimentally supported relationships; dashed arrows denote hypothesised relationships.

Upon synthesising the evidence, we propose a triangular pathogenic model in which OPMDs, periodontitis and immunosenescence form a mutually reinforcing axis (Figure 2). Periodontitis drives local and systemic immunosenescence through sustained inflammatory signalling, PD-1/PD-L1 upregulation, and myeloid skewing. Immunosenescence then impairs immune surveillance in OPMDs, reducing the clearance of senescent and dysplastic cells, and promoting a chronic, SASP-driven inflammatory microenvironment. This SASP-driven inflammation, in turn, fuels the progression of periodontitis and drives the malignant transformation of OPMDs through DNA damage, epigenetic alterations, and the activation of oncogenic pathways. This triangular relationship creates a positive feedback loop: periodontitis accelerates immunosenescence, immunosenescence impairs OPMD immune surveillance, and the resulting chronic inflammation sustains the progression of both periodontitis and OPMDs.

### 4.5. Mechanistic Pathways Linking Periodontitis to Oscc via Immunosenescence

Several specific molecular pathways illustrate how periodontitis-driven immunosenescence may promote OSCC development (Table 1).

The Wnt/β-catenin pathway: Wnt ligands Wnt3, Wnt3a, Wnt5b, and Wnt7b are upregulated in both periodontitis and oral carcinogenesis. This pathway plays a key role in OPMD-to-OSCC transformation, particularly in lesions with histological dysplasia. Immunosenescence may enhance Wnt signalling through SASP-mediated cytokine release, creating a permissive environment for β-catenin activation.

The NF-κB and STAT3 pathways: Pro-inflammatory cytokines that engage NF-κB signalling—TNF-α, IL-1, IL-6, and IL-8—are elevated in the saliva of OPMD patients. These cytokines activate STAT3 and other oncogenic pathways that induce tumour stem cell properties. Chronic periodontitis sustains this inflammatory signalling, while immunosenescence amplifies it through SASP.

The p53 and p16^INK4A^ pathways: Senescence markers p16^INK4A^ and p21 are upregulated in oral mucosa exposed to carcinogenic stimuli. Notably, increased p16^INK4A^ protein expression is a canonical marker of senescence, whereas epigenetic silencing of p16^INK4A^ by promoter hypermethylation—frequently observed in oral leukoplakia—is instead associated with escape from senescence and malignant progression; protein expression, promoter methylation, and functional activity of the p16^INK4A^–Rb axis should therefore be distinguished rather than treated as equivalent senescence readouts. *TP53* mutations, which accumulate with age and DNA damage, not only disrupt cell-cycle regulation but also activate PD-1/PD-L1 immune evasion pathways and promote epithelial–mesenchymal transition.

The cGAS-STING pathway: *P. gingivalis* OMVs suppress cGAS-STING innate immune signalling by disrupting IRF3–STING and IRF3–TBK1 interactions, impairing IFN-β production and limiting NK cell and dendritic cell recruitment to the tumour microenvironment—a novel mechanism by which periodontal pathogens directly undermine innate immune surveillance.

The phosphoinositide 3-kinase (PI3K)/Akt/mTOR pathway: This pathway integrates growth factor signalling with cellular metabolism and senescence regulation. In periodontitis, PI3K/Akt activation promotes inflammatory cytokine production and alveolar bone resorption. In immunosenescence, mTOR hyperactivity drives SASP translation and impairs autophagy, while in OSCC it supports proliferation, survival, and metabolic reprogramming. Rapamycin and other mTOR inhibitors have shown senomorphic effects by suppressing SASP, suggesting this pathway as a convergent therapeutic target across all three conditions.

### 4.6. Alternative Interpretations and Conflicting Evidence

While the evidence presented above supports the immune-senescent switch model, several alternative interpretations and conflicting findings also deserve consideration. Firstly, the link between periodontitis and OSCC could be distorted by shared risk factors. Although meta-analyses report significant associations after adjustment, the magnitude of the independent effect is still being debated. Some large cohort studies and systematic reviews have found that the association between periodontitis and oral cancer attenuates substantially after comprehensive adjustment for smoking, alcohol consumption, and socioeconomic status. This raises questions about whether periodontitis is a true independent cause of oral cancer or whether it is primarily a marker of cumulative exposure to shared risk factors [8,9].

Secondly, the role of senescence in tumour suppression versus promotion is context-dependent and not universally accepted. In certain tumour models, senescent stromal cells have been shown to suppress rather than promote neighbouring tumour cell proliferation depending on the SASP composition and the immune context. The idea that senescence always “flips” from protective to harmful may oversimplify the more complex biological reality. Furthermore, transient senescence plays essential physiological roles in wound healing and tissue homeostasis in the oral mucosa. Indiscriminately eliminating senescent cells could theoretically impair these protective functions [54].

Thirdly, the clinical translation of senolytics from the treatment of HNSCC to the interception of OPMDs remains speculative. The COIS-01 trial demonstrated efficacy in treating established, resectable HNSCC, but not OPMDs. Whether senolytics can prevent malignant transformation in premalignant lesions, where senescent cells may still exert tumour-suppressive effects, remains entirely untested. In theory, premature clearance of senescent cells in early OPMDs could remove a protective barrier and accelerate malignant progression. Finally, although senolytic treatment with dasatinib plus quercetin improved physical function and extended lifespan in aged mice [55], subsequent programmes have yielded mixed results: UNITY Biotechnology’s phase 2 trial of UBX1325 (a BCL-xL inhibitor) for diabetic macular oedema did not meet all endpoints, and efficacy has varied with dosing, timing, and genetic background. These findings caution against overgeneralising preclinical senolytic efficacy and emphasise the need for rigorous, indication-specific clinical validation.

Fourthly, even the microbial evidence is not uniformly supportive. Chander et al. reported that intratumoural *Fusobacterium* detectability was associated with better overall and disease-specific survival in two independent HNSCC cohorts, and that both *F. nucleatum* and *F. periodonticum* were directly toxic to OSCC cell lines in vitro, an effect mediated primarily by the bacterial secretome [56]. These findings complicate the prevailing view of *F. nucleatum* as a uniformly tumour-promoting organism and caution against extrapolating from colorectal cancer paradigms; they suggest that the net effect of periodontal pathogens on oral carcinogenesis may depend on context—bacterial strain, load, anatomical niche, and host immune state—and they qualify the microbial vertex of our model.

Finally, it is important to distinguish association, biological plausibility, and causal mediation. The epidemiological studies reviewed above establish association; the mechanistic studies establish biological plausibility; but neither demonstrates that immune-cell senescence mediates the effect of periodontitis on oral carcinogenesis. Several alternative explanations remain viable: periodontal pathogens may act directly on epithelial cells; chronic inflammation may promote carcinogenesis through senescence-independent routes; shared exposures, such as tobacco and alcohol, may drive both conditions; and tumour development may itself alter the oral microbiota. Moreover, adoptive-transfer experiments demonstrate the systemic pathogenic potential of periodontitis-induced senescent T cells, but they do not model the physiological trafficking of immune cells from periodontal sites to OPMDs. The decisive test of the proposed model would be to show that selective interference with immune-cell senescence—for example, the targeted clearance or rejuvenation of senescent immune cells—specifically attenuates periodontitis-associated oral carcinogenesis; such experiments have not yet been performed.

### 4.7. Distinguishing Established Evidence from the Proposed Model: A Critical Appraisal

Because the immune-senescent switch is a hypothesis rather than an established mechanism, we summarise here how each link in the proposed chain is currently supported. (i) The epidemiological association between periodontitis and oral cancer is supported by meta-analytic data (adjusted OR 2.32; 95% CI: 1.71–3.15) [5]; this constitutes established, though observational, evidence, and causality cannot be inferred because of residual confounding by tobacco, alcohol, and socioeconomic factors [8,9]. (ii) The capacity of periodontitis to accelerate immune senescence is supported by direct experimental evidence in murine models—including increased SA-β-gal activity, SASP cytokine production and the systemic transfer of senescent CD4+ T cells [25,26]—but corroborating human data remain correlational. (iii) The contribution of immunosenescence to cancer development and immunotherapy resistance is well established in oncology generally [15,16], and oral-specific evidence (e.g., immunosenescence signatures in non-responsive HNSCC [16,19]) is accumulating, though it remains indirect with respect to OPMD progression. (iv) By contrast, the “switch” itself—a discrete or threshold-like transition of senescence from tumour-suppressive to tumour-promoting in the oral mucosa—has not been demonstrated directly in any model; it is a conceptual extrapolation from the dual roles of senescence documented in other contexts [22,42] and from the temporal pattern of T-cell exhaustion observed in the 4NQO model [46]. (v) Finally, clinical evidence that targeting immunosenescence improves outcomes is currently limited to a single, small, single-arm trial in established HNSCC [19] and cannot yet be extrapolated to OPMD interception.

We therefore regard the immune-senescent switch as a testable framework that organises existing evidence and generates specific, falsifiable predictions—for example, that biomarkers of immunosenescence will precede histological progression in longitudinal OPMD cohorts, and that periodontal intervention will measurably attenuate systemic senescent T-cell burden—rather than as an established description of oral carcinogenesis.

## 5. Therapeutic Implications: Targeting the Immune-Senescent Switch

If immunosenescence indeed links periodontitis and the malignant transformation of OPMDs, then targeting immunosenescence could be a promising way to prevent oral carcinogenesis. The following sections review emerging senotherapeutic approaches (Table 2), emphasising landmark clinical evidence and precise reprogramming strategies, and distinguishing between established cancer treatment and the more speculative interception of OPMDs [54,55].

### 5.1. Senolytics: Clearing Senescent Cells

Senolytics selectively eliminate senescent cells. Preclinical studies have shown promising results for B-cell lymphoma 2 (BCL-2) inhibitors such as ABT-263 (navitoclax) and tyrosine kinase inhibitors such as dasatinib, which is often combined with quercetin. In head and neck cancer models, combining dasatinib and quercetin with PD-1 blockade has been shown to significantly reduce tumour burden and extend survival compared with PD-1 inhibitors alone. This approach has also been found to restore naïve T-cell function and reverse immune ageing markers [19].

The COIS-01 trial (NCT05724329) represents an early clinical test of targeting immunosenescence-driven immunotherapy resistance [19]. This investigator-initiated, single-arm, phase 2 trial was conducted by a collaborative team from Sun Yat-sen Memorial Hospital, Sun Yat-sen University, the Guangzhou National Laboratory and Harvard Medical School. It built upon insights from the preceding OOC-001 phase 2 trial.

In the OOC-001 trial, 51 patients with resectable HNSCC received neoadjuvant chemoimmunotherapy (sintilimab plus carboplatin and nab-paclitaxel). Although 23 patients achieved a pathological complete response, some patients derived no benefit. Single-cell multi-omics profiling of tumours and blood revealed that non-responding tumours exhibited prominent immunosenescence features (see Section 2.1), alongside elevated expression of immunosenescence-related genes in T- and B-cell subsets—directly linking poor response to immunosenescence within the tumour microenvironment [19].

Encouraged by these findings, the researchers launched the COIS-01 trial to test the specific combination of tislelizumab with the senolytic regimen dasatinib plus quercetin in patients with established HNSCC. Twenty-four patients with resectable HNSCC received the neoadjuvant regimen of tislelizumab (anti-PD-1), dasatinib and quercetin. This regimen achieved a major pathological response (MPR) rate of 33.3% (95% CI: 16.6–54.7%) [19]. For context, pathological response rates reported for neoadjuvant anti-PD-1 monotherapy have been considerably lower: in the CheckMate 358 trial, only 5.9% of centrally evaluable human papillomavirus (HPV)-positive tumours achieved an MPR and no pathological complete responses were observed [57], while objective radiographic response rates to anti-PD-1 monotherapy in recurrent/metastatic disease have ranged from approximately 13% to 19% [58,59]. However, these comparisons are indirect and must be interpreted with caution, given differences in trial design, patient populations, and endpoints (pathological versus radiographic response), and given the small, single-arm nature of COIS-01 (*n* = 24), which lacked a concurrent control group. The toxicity profile was favourable: only one patient (4.2%) experienced grade 3–4 adverse events, compared with more than half of patients in the OOC-001 chemoimmunotherapy cohort (a cross-trial comparison, to be interpreted with caution). In OOC-001, the most common treatment-related adverse events were alopecia (75%), asthenia/fatigue (53%), and nausea (49%), whereas in COIS-01 the corresponding incidences were 0%, 25%, and 16.7%, respectively.

Immunohistochemical analysis before and after treatment revealed upregulated CCR7 expression in naïve T cells post-treatment, indicating mitigation of immunosenescence-related features and restoration of adaptive immune capacity. To date, there have been no recurrences or deaths in the COIS-01 cohort, although follow-up remains short and the durability of these responses is not yet established. Taken together, these findings suggest preliminary activity and tolerability of this specific tislelizumab–dasatinib–quercetin combination in established HNSCC. Because COIS-01 was small and single-arm, however, it cannot establish the incremental contribution of senolysis over PD-1 blockade alone, and comparisons between neoadjuvant pathological response rates and radiographic response rates reported in recurrent or metastatic settings do not provide an adequate basis for comparative conclusions; randomised controlled trials are required.

Importantly, COIS-01 was conducted in patients with resectable HNSCC, not OPMDs. While these results provide proof of concept that targeting immunosenescence can enhance the efficacy of immunotherapy, extrapolation to OPMD interception remains hypothetical. As discussed in Section 4.6, whether senolytics can prevent malignant transformation in premalignant lesions remains untested and requires dedicated clinical trials. Moreover, the COIS-01 results pertain to the specific tislelizumab–dasatinib–quercetin regimen and should not be generalised to other senolytic agents, such as fisetin or navitoclax, or to OPMD prevention.

### 5.2. First-Generation Senolytics: Limitations and Next-Generation Strategies

Despite showing promise in preclinical studies, first-generation senolytics face significant challenges in translation. For example, navitoclax (ABT-263), a potent BCL-2/BCL-xL/BCL-w inhibitor, causes dose-limiting thrombocytopenia in 44–50% of patients because platelets rely on BCL-xL for survival. This on-target toxicity has prompted the development of next-generation strategies, such as platelet-sparing BCL-xL inhibitors (e.g., UBX1325), p16^INK4A^-targeted suicide gene delivery systems (the Oisín lipid nanoparticle (LNP) platform), and ferroptosis-based senolysis (RLS-1496, a glutathione peroxidase 4 (GPX4) modulator). In dermatological applications, RLS-1496 achieved a 20% reduction in epidermal thickness with a robust safety profile, suggesting that localised, targeted senolysis may offer the most viable near-term translational path.

Fisetin, a natural polyphenol that targets the BCL-xL and PI3K/Akt/mTOR pathways, has demonstrated greater senolytic potency than quercetin in certain preclinical models. Human trials have progressed with high daily doses (up to 20 mg/kg/day) and exceptional safety profiles. The ongoing TROFFi phase 2 study is investigating whether high-dose fisetin can mitigate the decline in physical function experienced by postmenopausal breast cancer survivors following chemotherapy-induced senescence. A novel galactomannan formulation derived from fenugreek seeds has increased fisetin bioavailability by up to 27-fold in human studies, which could overcome a significant obstacle to clinical translation.

However, the lifespan extension observed with senolytic treatment in preclinical studies [55] has not been uniformly replicated across independent testing programmes, highlighting the potential for discrepancies to arise due to differences in dosing, timing, genetic background, or strain. These discrepancies underscore the need for species-specific validation and carefully designed clinical trials. In the context of oral cancer, fisetin is an intriguing prospect, as it can be delivered locally via oral mucosal application, which could minimise systemic toxicity while achieving therapeutic concentrations in the oral microenvironment. Further preclinical research in models of periodontitis-associated oral carcinogenesis is required to evaluate this localised delivery strategy.

### 5.3. Senomorphics: Modulating the Sasp

Senomorphics (also known as senostatics) modulate the SASP without eliminating senescent cells. Rapamycin, an mTOR inhibitor, downregulates the SASP by inhibiting the mTORC1 pathway, which is essential for the translation of SASP factors. Janus kinase (JAK) inhibitors, such as ruxolitinib, reduce systemic inflammation by blocking JAK/STAT signalling downstream of SASP cytokines. Metformin and aspirin have also demonstrated SASP-modulating potential through AMP-activated protein kinase (AMPK) activation and NF-κB inhibition, respectively. Agents that stimulate the immune system, such as Toll-like receptor 5 (TLR5) or TLR7 agonists, and geroprotective agents, such as rapamycin, metformin, and nicotinamide adenine dinucleotide (NAD+) precursors, have been proposed to address immunosenescence. This approach may be particularly relevant for patients with periodontitis, in whom chronic SASP-driven inflammation sustains both periodontal destruction and OPMD progression. However, the non-specific nature of these agents and their potential for systemic immunosuppression remain significant concerns.

### 5.4. Immune-Based Clearance and Rejuvenation

There is an emerging focus on immunotherapeutic strategies to enhance senescent cell clearance. Chimeric antigen receptor T-cell therapies that target senescence-associated surface proteins, such as the urokinase-type plasminogen activator receptor (uPAR), have been validated in mouse models. Novel “senescent cell–NK cell” coupling peptides can clear therapy-induced senescent cells while reducing toxicity. In the context of periodontitis-driven immunosenescence, strategies to rejuvenate the immune system—including checkpoint modulation, cytokine therapy, and adoptive cell transfer—may restore the capacity to clear senescent cells and eliminate premalignant clones [54]. Other emerging strategies, such as senolytics, epigenetic modulators (e.g., histone deacetylase inhibitors), and metabolic interventions (e.g., spermidine and nicotinamide mononucleotide), provide further potential for rejuvenating aged immunity. Histone deacetylase inhibitors, such as trichostatin A and suberoylanilide hydroxamic acid (SAHA), trigger histone hyperacetylation, thereby reducing cancer stem cell populations and sensitising HNSCC cells to chemotherapeutic agents, such as cisplatin. Cyclin-dependent kinase 4/6 (CDK4/6) inhibition induces senescence in malignant cells and exhibits synergistic therapeutic effects in melanoma when combined with immunotherapy, thereby overcoming resistance to treatment [43].

### 5.5. From Broad-Spectrum Senolysis to Precision Reprogramming: An Integrated Framework

**Figure 3:**
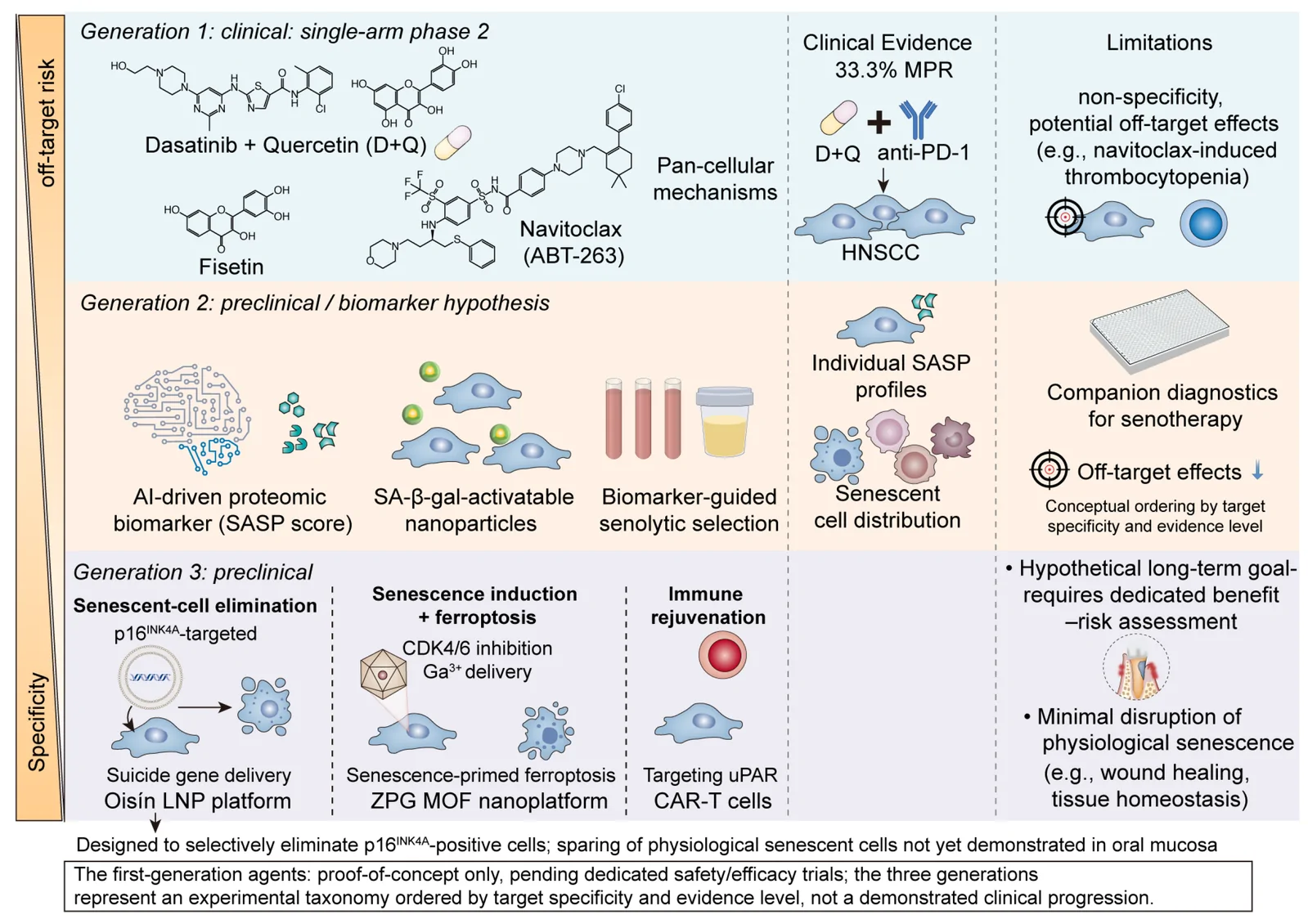
Three-generation framework for senotherapeutic development in oral cancer prevention and therapy. This conceptual framework illustrates the evolutionary trajectory of senotherapeutic strategies from broad-spectrum senolysis to precision reprogramming. First-generation senolytics—dasatinib plus quercetin, navitoclax, and fisetin—represent foundational broad-spectrum agents that eliminate senescent cells through pan-cellular mechanisms. The single-arm phase 2 COIS-01 trial provided preliminary evidence of clinical activity in HNSCC, with a 33.3% MPR and favourable toxicity. However, their non-specific nature and off-target effects limit broad applicability. Second-generation strategies emphasise targeted delivery and biomarker-guided selection. The SASP Score could enable quantification of individual SASP profiles and, prospectively, personalised senotherapeutic selection (not yet validated in oral cohorts). Nanoparticle-based targeted delivery systems, such as SA-β-gal-activatable bioorthogonal photosensitisers, enable selective drug retention in senescent tumour cells, overcoming efflux-mediated resistance and reprogramming SASP towards an immunogenic profile. Third-generation precision reprogramming aims for molecular-level specificity by exploiting senescence-induced vulnerabilities rather than merely eliminating senescent cells. The Oisín LNP platform delivers p16^INK4A^-targeted suicide gene systems designed to achieve selective elimination of p16-positive senescent cells; whether physiological senescent cells are spared remains to be established. The ZPG metal–organic framework nanoplatform co-delivers a CDK4/6 inhibitor (palbociclib) and a ferroptosis inducer (Ga^3+^), inducing “senescence-primed ferroptosis” through redox reprogramming. The three generations represent an experimental taxonomy ordered by target specificity and evidence level, not a demonstrated clinical progression. The stratified clinical translation pathway shows: first-generation agents as initial proof-of-concept in high-risk OPMD patients with concurrent periodontitis (pending safety trials); second-generation biomarker-guided strategies enabling personalised selection; and third-generation precision reprogramming potentially achieving the specificity that would be required for long-term preventive interventions; such preventive use remains hypothetical and requires dedicated benefit–risk assessment.

The field is rapidly evolving from broad-spectrum senolysis towards precision reprogramming. Building on a recent senotherapeutic taxonomy [63], we present a three-generation classification as an experimental framework that groups strategies by target specificity and evidence level (Figure 3); the generational sequence reflects conceptual development rather than a demonstrated clinical progression. These generations also encompass conceptually distinct strategies—senescent-cell elimination, immune rejuvenation, and senescence induction coupled to ferroptosis—which should not be conflated under a single mechanistic umbrella.

First-generation senolytics (dasatinib + quercetin, navitoclax, and fisetin) are foundational, broad-spectrum agents that eliminate senescent cells through pan-cellular mechanisms. Although the COIS-01 trial provided preliminary, single-arm evidence of activity for one such combination in HNSCC (see Section 5.1), their non-specific nature and off-target effects (e.g., navitoclax-induced thrombocytopenia; see Section 5.2) limit their applicability. These agents are therefore best suited to patients with a high systemic senescent-cell burden and limited comorbidities.

Second-generation strategies emphasise targeted delivery and biomarker-guided selection. The SASP Score, an artificial intelligence (AI)-driven proteomic biomarker, could in future enable patient stratification by quantifying individual SASP profiles, potentially allowing clinicians to match patients with the most appropriate senotherapeutic modality; this treatment-selection application remains a hypothesis that has not yet been validated in oral cancer cohorts. Nanoparticle-based targeted delivery systems, such as SA-β-gal-activatable bioorthogonal photosensitisers, enable selective drug retention in senescent tumour cells, overcoming efflux-mediated resistance. These approaches can degrade PD-L1, inhibit the indoleamine 2,3-dioxygenase–kynurenine (IDO-Kyn) pathway and reprogram the SASP towards an immunogenic profile. This represents a conceptual shift from systemic senolytic administration to precision-targeted elimination.

Third-generation precision reprogramming aims for molecular-level specificity. The Oisín LNP platform delivers p16^INK4A^-targeted suicide gene systems, which are designed to achieve the selective elimination of p16-positive senescent cells; however, direct evidence that such targeting spares physiological senescent cells involved in wound healing and tissue homeostasis in the oral mucosa is still required. A novel, metal–organic framework-based nanoplatform (ZPG) co-delivers a CDK4/6 inhibitor (palbociclib) and a ferroptosis inducer (gallium ions, Ga^3+^) to enhance anti-tumour efficacy in OSCC [60]. In OSCC cells with CDK4/6 hyperactivity, palbociclib selectively blocks cell-cycle progression and induces robust senescence. This leads to the downregulation of the anti-ferroptosis factors GPX4 and glutathione (GSH), and the upregulation of the pro-ferroptosis factor acyl-CoA synthetase long-chain family member 4 (ACSL4) with concomitant Fe^2+^ accumulation. This redox reprogramming compromises cellular antioxidant defences and promotes lipid peroxidation, thereby sensitising senescent cells to ferroptosis. This “senescence-primed ferroptosis” strategy is an example of a third-generation approach that exploits senescence-induced vulnerabilities rather than merely eliminating senescent cells.

The clinical translation of this framework requires a stratified approach. First-generation agents could initially provide proof of concept in high-risk OPMD patients with concurrent periodontitis, provided that dedicated safety and efficacy trials are conducted. Second-generation, biomarker-guided strategies could enable personalised selection based on SASP profiles and senescent-cell burden. Third-generation, precision reprogramming may ultimately achieve the specificity required for long-term preventive interventions in the oral cavity, where transient senescence plays an essential role in wound healing and tissue homeostasis. Preventive use in OPMDs requires a distinct benefit–risk assessment, because the target population is healthier, the intervention horizon is longer than in oncology, and interference with physiological senescence could impair oral wound healing.

### 5.6. Periodontal Therapy as a Potential Modulator of Immunosenescence

One intriguing implication of the immunosenescence–periodontitis–OSCC axis is that effective periodontal therapy may modulate immunosenescence itself. By reducing the burden of chronic inflammation, periodontal treatment could slow or even reverse certain aspects of immunosenescence, thereby lowering the risk of malignant transformation in OPMDs. However, this hypothesis requires direct testing. Currently, no randomised controlled trial or longitudinal cohort study has demonstrated that periodontal treatment reduces the incidence of oral cancer or the rate of malignant transformation of OPMDs.

Periodontal treatment reduces serum CRP and IL-6 levels within two months, providing indirect evidence for its potential role in modulating systemic inflammaging [11]. A systematic review by D’Aiuto et al. showed that periodontal treatment consistently improves systemic inflammatory biomarkers, with significant reductions in CRP and IL-6 across multiple randomised controlled trials [61]. Furthermore, intensive non-surgical periodontal therapy in patients with severe periodontitis has been shown to significantly reduce serum CRP, IL-6, and TNF-α levels, providing clinical evidence that periodontal intervention can modulate the systemic inflammatory milieu that drives immunosenescence [62]. These findings support the hypothesis that periodontal therapy may serve as a practical and accessible intervention to mitigate inflammaging. Nevertheless, we caution against overinterpreting these biomarker changes as proof of cancer prevention. The relationship between cancer and periodontitis highlights the importance of integrating oral health into oncological care and immunotherapy management. However, periodontal therapy should currently be considered a standard oral health maintenance treatment; any systemic anti-cancer benefit remains hypothetical.

## 6. Gaps, Challenges, and Future Directions

Despite the accumulation of evidence in support of the immunosenescence–periodontitis–OSCC axis, critical gaps persist. We have organised these gaps into three hierarchical levels: fundamental scientific questions, translational barriers, and methodological limitations.

### 6.1. Fundamental Scientific Questions

Firstly, the role of senescent immune cells within the oral tumour microenvironment is not well understood. Although senescent T cells, macrophages, and NK cells accumulate with age and chronic inflammation, their specific contributions to OPMD progression and malignant transformation require direct investigation. Secondly, the spatial distribution of senescent immune cells within the gingival lamina propria, adjacent OPMD tissues, and the transition zone between periodontitis and dysplasia is unknown.

Thirdly, the causal pathways linking oral cancer to systemic disease in older adults are not fully understood. While periodontitis and OSCC share epidemiological associations, establishing causality—and specifically demonstrating that periodontitis-driven immunosenescence directly promotes OSCC—demands rigorous mechanistic studies. Multi-omics approaches, longitudinal microbiome profiling, immune phenotyping (including single-cell and spatial methods), and systems immunology now enable the mapping of host–microbe–immune interactions at a mechanistic level.

Fourthly, the temporal dynamics of the immune-senescent switch require clarification. Does the switch represent a discrete event or a gradual continuum? At what point does senescence transition from protective to deleterious in the OPMD microenvironment? Answering these questions will require longitudinal sampling of OPMD lesions with integrated molecular and immune phenotyping.

### 6.2. Translational Barriers

There is a lack of biomarkers capable of distinguishing between pro-tumourigenic and anti-tumourigenic senescence. Developing such biomarkers is essential for risk stratification and monitoring responses to senotherapeutic interventions. In this context, it is useful to distinguish diagnostic biomarkers (malignant-versus-benign discrimination), prognostic biomarkers (transformation-risk prediction), treatment-predictive biomarkers (response to a specific senotherapeutic), and pharmacodynamic biomarkers (target engagement). Most candidate senescence biomarkers in oral oncology, including IL-6 and CXCL1, have so far been evaluated only as diagnostic markers; moreover, inflammatory cytokines are not senescence-specific, underscoring the need for composite, senescence-anchored biomarker panels. OPMDs could be dynamically monitored using liquid biopsies, with the loss of cellular senescence serving as a transformation biomarker. While the SASP Score and other AI-driven proteomic biomarkers show promise, validation in oral cancer cohorts is urgently needed.

Quantitative evidence supports the potential of SASP factors as biomarkers in oral cancer. Analysis of 72 cancerous and 64 normal HNSCC tissues revealed significantly elevated transcript levels of IL-6 (*p* < 0.0001), IL-1β (*p* < 0.0001), CXCL1 (*p* < 0.0001), and TNF-α (*p* = 0.0015) in cancerous tissues. IL-6 and CXCL1 demonstrated robust diagnostic performance, with respective receiver operating characteristic (ROC) area under the curve (AUC) values of 0.8816 (sensitivity 84%, specificity 72%) and 0.8812 (sensitivity 86.67%, specificity 86.96%) [23]. Importantly, these AUC values concern the discrimination of malignant from normal tissues in a head and neck cohort (oral-cancer subgroup), not the prospective prediction of OPMD transformation; the original study also found no correlation between tissue CXCL1 transcript levels and serum protein levels, which limits direct extrapolation to liquid-biopsy applications. These findings identify SASP factors as quantifiable molecular signatures that distinguish malignant from normal oral tissues, and motivate—rather than establish—their evaluation as companion diagnostics for senotherapeutic interventions. However, prospective validation in longitudinal OPMD cohorts is required to ascertain whether these biomarkers can predict malignant transformation prior to evident histological progression.

The optimal approach to targeting immunosenescence in the OPMD setting is unclear. Should we eliminate senescent cells using senolytics, modulate the SASP using senomorphics, rejuvenate the immune system, or combine these approaches? While the “chemo-induced senescence + senolytic clearance” sequential strategy has shown promise in cancer treatment, its application to OPMD requires careful evaluation. Although the COIS-01 trial provides a preliminary precedent for established HNSCC, the optimal type, dosage and duration of senolytics to be used in combination with immune checkpoint inhibitors for OPMD patients remain undefined.

The balance between therapeutic benefit and harm must be carefully considered, particularly in older patients with impaired oral tissue function. Senescence plays essential roles in wound repair, tissue homeostasis, and tumour suppression. The indiscriminate elimination of senescent cells could therefore impair these protective functions, particularly in the oral mucosa where tissues are subject to constant mechanical, microbial, and environmental challenges. The impact of senolytic therapy on oral mucosal regeneration and wound healing—processes dependent on transient senescence—requires careful evaluation.

### 6.3. Methodological Limitations

Most preclinical studies of senescence and oral cancer have used young, healthy animal models. However, given that both periodontitis and OSCC predominantly affect older adults, aged animal models that replicate the human condition are urgently needed. Studies using naturally aged mice and *Ercc1*-deficient mice (which exhibit premature ageing) have demonstrated that senolytics can enhance the efficacy of immunotherapy in multiple solid tumours by mitigating immunosenescence [19]. However, models incorporating periodontitis as a risk factor for oral carcinogenesis have yet to be developed.

Recent evidence supports the feasibility of such integrated models. For example, Li et al. demonstrated that ligature-induced periodontitis in mice accelerates CD4+ T-cell senescence in splenic lymphocytes, resulting in increased SA-β-gal activity and SASP cytokine expression. Furthermore, they showed that adoptive transfer of these senescent T cells exacerbated collagen antibody-induced arthritis in recipient nude mice [26]. This study provides a validated experimental framework for investigating the causal relationship between periodontitis-driven immunosenescence and systemic disease progression, which could be adapted to incorporate oral carcinogenesis endpoints.

The integration of multi-omics approaches, such as spatial transcriptomics, single-cell immune profiling, and microbiome sequencing, offers unprecedented opportunities to analyse the spatiotemporal dynamics of the immune-senescent switch in the oral microenvironment. Spatial transcriptomics has revealed that tumour microenvironment composition varies drastically between tumour core and invasive front, with distinct cancer-associated fibroblast and immune cell distributions influencing therapeutic response. Future studies should leverage these technologies to map the precise anatomical and temporal coordinates of senescence-driven immune evasion in OPMDs.

Developing AI-powered prognostic models to predict OPMD malignant transformation is a promising translational approach. Machine learning algorithms that integrate clinical, molecular, and microbiome data could enable precise risk stratification and guide senotherapeutic interventions. Incorporating senescence biomarkers (p16^INK4A^ and SASP factors), microbiome signatures (abundance of *F. nucleatum* and *P. gingivalis*) and immune phenotypes (PD-1/PD-L1 expression and T-cell exhaustion markers) into predictive models could transform the management of OPMDs from passive surveillance to active interception.

## 7. Conclusions

The ageing global population, the high prevalence of periodontitis, and the increasing burden of OSCC require a better understanding of the links between these issues. This review summarises the growing body of evidence suggesting that immunosenescence may constitute a mechanistic link between periodontitis-driven chronic inflammation and OSCC malignant transformation, while emphasising that this link remains a hypothesis supported by evidence of varying strength.

We propose—explicitly as a testable hypothesis rather than an established mechanism—an “immune-senescent switch” model in which periodontitis accelerates immunosenescence within the oral microenvironment, progressively eroding local immune surveillance. In early OPMDs, senescence is proposed to act as a tumour-suppressive barrier, enforcing cell-cycle arrest and recruiting immune cells to clear damaged cells. However, chronic periodontitis-driven immunosenescence is hypothesised to flip this switch, resulting in the accumulation of senescent immune cells, sustained SASP-driven inflammation, dysregulation of immune checkpoint pathways, and impaired clearance of senescent and premalignant cells. This transition is governed by three interdependent parameters: senescent-cell burden, SASP composition, and immune effector integrity. On this hypothesis, periodontitis would accelerate the crossing of this threshold across all three parameters.

The COIS-01 trial has provided the first clinical proof of concept that targeting immunosenescence can enhance the efficacy of immunotherapy while reducing toxicity in established HNSCC, although these results derive from a small, single-arm study, and extrapolation to OPMD interception remains hypothetical and requires dedicated investigation. This paradigm shift—from viewing cancer as purely neoplastic to addressing the ageing immune microenvironment—carries profound implications for oral cancer prevention. For OPMD patients with concurrent periodontitis, integrated management strategies combining periodontal therapy, senolytic/senomorphic interventions, and immune checkpoint modulation could intercept oral carcinogenesis before malignant transformation becomes irreversible. However, we emphasise that these strategies are still speculative and require rigorous preclinical and clinical validation. If validated, targeting the immune-senescent switch could, in the long term, help transform the management of OPMDs from surveillance and late intervention towards active prevention and early interception; at present, this remains a research agenda rather than a clinical recommendation.

## Figures and Tables

**Table 1:** Key molecular pathways linking periodontitis, immunosenescence, and OSCC.

Pathway	Role in Periodontitis	Role in Immunosenescence	Role in OSCC Development	Key Mediators/Therapeutic Targets	Key References
Wnt/β-catenin	Wnt ligands upregulated in periodontal inflammation; promotes alveolar bone loss via RANKL induction	SASP cytokines enhance Wnt signalling in neighbouring cells; creates pro-inflammatory feed-forward loop	Key driver of OPMD→OSCC transformation; promotes EMT, stemness, and invasion	β-catenin, TCF/LEF, MYC, Cyclin D1/Porcupine inhibitors, anti-Fzd antibodies	[42]
NF-κB/STAT3	Sustained activation by periodontal pathogens (LPS, gingipains); drives inflammatory cytokine production	Amplified by SASP from senescent immune cells; promotes chronic inflammaging via IL-6-STAT3 axis	Promotes tumour stem cell properties, proliferation, survival, and angiogenesis; mediates immunosuppressive TME	IL-6, IL-1β, TNF-α, STAT3, NF-κB (p65)/JAK inhibitors (ruxolitinib), STAT3 inhibitors	[29,43]
PD-1/PD-L1	Upregulated in periodontitis; PD-1+ T cells accumulate; PD-L1 expressed on macrophages and epithelial cells	T-cell exhaustion and impaired immune surveillance; PD-1+ senescent CD8+ T cells accumulate in TME	Immune evasion and poor prognosis; PD-L1+ TAMs and tumour cells suppress cytotoxic T-cell function	PD-1, PD-L1, IFN-γ, CD8+ T cells/Anti-PD-1/PD-L1 antibodies (tislelizumab, pembrolizumab, nivolumab)	[29,30,31,44,45]
p53/p16^INK4A^	DNA damage from chronic inflammation; oxidative stress-induced mutations in periodontal tissues	Senescence marker accumulation (p16^INK4A^, p21); loss of p53 function impairs senescent cell clearance	Loss of cell-cycle control; EMT activation; genomic instability; TP53 mutations promote PD-L1 upregulation	p16^INK4A^, p21, p53, Rb, MDM2/MDM2 inhibitors, p53 reactivators	[42,46,44]
cGAS-STING	*P. gingivalis* OMVs disrupt STING–TBK1–IRF3 interactions; impair IFN-β production	Impaired innate immune sensing in senescent microenvironment; reduced NK and DC recruitment	Reduced IFN-β and CXCL9/10 production; impaired anti-tumour immunity; immune evasion via attenuated type I IFN signalling	cGAS, STING, TBK1, IRF3, IFN-β, CXCL9/10/STING agonists (ADU-S100, MSA-2; exploratory, context-dependent—see text and footnote), TBK1 modulators	[36,39,41]
PI3K/Akt/mTOR	PI3K/Akt activation promotes inflammatory cytokine production and alveolar bone resorption	mTOR hyperactivity drives SASP translation and impairs autophagy during immune ageing	Contributes to OSCC progression, survival, and metabolic reprogramming	Akt, mTOR, FoxO1, PI3K/mTOR inhibitors (rapamycin, everolimus), PI3K inhibitors	[43]

Akt: protein kinase B; cGAS: cyclic GMP-AMP synthase; CXCL9/10: C-X-C motif chemokine ligands 9 and 10; DC: dendritic cell; EMT: epithelial–mesenchymal transition; FoxO1: forkhead box protein O1; Fzd: Frizzled; IFN: interferon; IL: interleukin; IRF3: interferon regulatory factor 3; JAK: Janus kinase; LPS: lipopolysaccharide; MDM2: murine double minute 2; mTOR: mechanistic target of rapamycin; MYC: MYC proto-oncogene; NF-κB: nuclear factor-κB; NK: natural killer; OMV: outer membrane vesicle; OPMD: oral potentially malignant disorder; OSCC: oral squamous cell carcinoma; PD-1: programmed cell death protein 1; PD-L1: programmed death-ligand 1; PI3K: phosphoinositide 3-kinase; RANKL: receptor activator of nuclear factor-κB ligand; Rb: retinoblastoma protein; SASP: senescence-associated secretory phenotype; STAT3: signal transducer and activator of transcription 3; STING: stimulator of interferon genes; TAM: tumour-associated macrophage; TBK1: TANK-binding kinase 1; TCF/LEF: T-cell factor/lymphoid enhancer-binding factor; TME: tumour microenvironment; TNF-α: tumour necrosis factor-α. STING agonists are listed as exploratory, preclinical agents; their activity is cell-type-, duration-, and context-dependent (see Section 3.5); The arrow (→) indicates the direction of progression from OPMD to OSCC.

**Table 2:** Therapeutic strategies targeting the immune-senescent switch.

Strategy	Mechanism	Evidence in OSCC/HNSCC	Clinical Trial Status	Key References
Senolytics	Selective elimination of senescent cells via BCL-2, tyrosine kinase, or PI3K/Akt/mTOR inhibition; restores naïve T-cell function	COIS-01 (phase 2): 33.3% MPR (95% CI: 16.6–54.7%), 4.2% grade 3–4 AEs; restored CCR7+ naïve T cells; no recurrence to date (short follow-up)	phase 2 (NCT05724329), single-arm, in HNSCC; OPMD trials not yet initiated	[19,57,58]
Senomorphics	Modulation of SASP without cell elimination; inhibits SASP translation or downstream signalling	Preclinical evidence in ageing models; SASP factor biomarker validation (IL-6 AUC 0.8816); reduces systemic inflammation via JAK/STAT blockade	Preclinical/early clinical	[23,24,43]
Immune checkpoint inhibitors	Restore T-cell function by blocking PD-1/PD-L1 interaction; reverses T-cell exhaustion	Enhanced efficacy when combined with senolytics (COIS-01); PD-L1 upregulated in periodontitis-associated OSCC; approved for HNSCC	Approved for HNSCC; phase 2 combinations ongoing	[2,19,29,30,59]
Immune rejuvenation	Restore aged immune cell function via CAR-T, cytokine modulation, or NAD+ metabolic intervention	CAR-T validated in lung adenocarcinoma and mouse models; NK cell coupling peptides in development; uPAR CAR-T in preclinical phase	Preclinical	[54,59]
Precision reprogramming	Targeted senescent cell elimination via p16^INK4A^-targeted suicide genes, SA-β-gal-activated prodrugs, or senescence-primed ferroptosis	ZPG platform demonstrated senescence-primed ferroptosis in OSCC cells: downregulates GPX4/GSH, upregulates ACSL4/Fe^2+^; selectively eliminates p16+ cells	Preclinical	[60]
Periodontal therapy	Reduce chronic inflammatory burden via mechanical debridement, antimicrobial therapy, and host modulation	Reduces serum CRP and IL-6 after 2 months in periodontitis patients; no direct evidence for OPMD malignant transformation prevention	Standard of care for periodontitis; hypothesis-generating for systemic inflammation modulation	[11,61,62]

ACSL4: acyl-CoA synthetase long-chain family member 4; AE: adverse event; AUC: area under the curve; BCL-2: B-cell lymphoma 2; CAR-T: chimeric antigen receptor T cell; CCR7: C-C chemokine receptor type 7; CI: confidence interval; CRP: C-reactive protein; GPX4: glutathione peroxidase 4; GSH: glutathione; HNSCC: head and neck squamous cell carcinoma; IL-6: interleukin-6; JAK/STAT: Janus kinase/signal transducer and activator of transcription; MPR: major pathological response; mTOR: mechanistic target of rapamycin; NAD+: nicotinamide adenine dinucleotide; NK: natural killer; OPMD: oral potentially malignant disorder; OSCC: oral squamous cell carcinoma; PD-1: programmed cell death protein 1; PD-L1: programmed death-ligand 1; PI3K: phosphoinositide 3-kinase; SA-β-gal: senescence-associated β-galactosidase; SASP: senescence-associated secretory phenotype; uPAR: urokinase-type plasminogen activator receptor; ZPG: metal–organic framework nanoplatform co-delivering palbociclib and gallium ions.

## Data Availability

No new data were created or analysed in this study. Data sharing is not applicable to this article.

## Abbreviations

The following abbreviations are used in this manuscript:

4NQO	4-Nitroquinoline-1-oxide
CDK4/6	Cyclin-dependent kinase 4/6
cGAS-STING	Cyclic GMP-AMP synthase–stimulator of interferon genes
CRP	C-reactive protein
CXCL1	C-X-C motif chemokine ligand 1
GPX4	Glutathione peroxidase 4
HNSCC	Head and neck squamous cell carcinoma
IFN-γ	Interferon-γ
IL	Interleukin
MMP	Matrix metalloproteinase
MPR	Major pathological response
mTOR	Mechanistic target of rapamycin
NF-κB	Nuclear factor-κB
NK	Natural killer
OMV	Outer membrane vesicle
OPMD	Oral potentially malignant disorder
OR	Odds ratio
OSCC	Oral squamous cell carcinoma
PD-1	Programmed cell death protein 1
PD-L1	Programmed death-ligand 1
PI3K	Phosphoinositide 3-kinase
SA-β-gal	Senescence-associated β-galactosidase
SASP	Senescence-associated secretory phenotype
SMD	Standardised mean difference
STAT3	Signal transducer and activator of transcription 3
TAM	Tumour-associated macrophage
TNF-α	Tumour necrosis factor alpha

## References

[1] Chi A.C., Day T.A., Neville B.W. (2015). Oral cavity and oropharyngeal squamous cell carcinoma—An update. CA Cancer J. Clin..

[2] Bernier J., Domenge C., Ozsahin M., Matuszewska K., Lefèbvre J.L., Greiner R.H., Giralt J., Maingon P., Rolland F., Bolla M. (2004). Postoperative irradiation with or without concomitant chemotherapy for locally advanced head and neck cancer. N. Engl. J. Med..

[3] Warnakulasuriya S., Kujan O., Aguirre-Urizar J.M., Bagan J.V., González-Moles M.Á., Kerr A.R., Lodi G., Mello F.W., Monteiro L., Ogden G.R. (2021). Oral potentially malignant disorders: A consensus report from an international seminar on nomenclature and classification, convened by the WHO Collaborating Centre for Oral Cancer. Oral Dis..

[4] Hanahan D. (2022). Hallmarks of cancer: New dimensions. Cancer Discov..

[5] Lin E.C., Chen J.H., Sitt S., Lin G.H., Wu C.Y., Wang C.W. (2026). The association between concurrent oral cancer and periodontal disease: A systematic review and meta-analysis. Quintessence Int..

[6] Tezal M., Sullivan M.A., Hyland A., Marshall J.R., Stoler D., Reid M.E., Loree T.R., Rigual N.R., Merzianu M., Hauck L. (2009). Chronic periodontitis and the incidence of head and neck squamous cell carcinoma. Cancer Epidemiol. Biomark. Prev..

[7] Xiao T., Hu G., Li T., Zhu X., Wang H., Zhu Z. (2025). Causal association between periodontitis and oral cancer: A two-sample Mendelian randomization study. Discov. Oncol..

[8] Javed F., Warnakulasuriya S. (2016). Is there a relationship between periodontal disease and oral cancer? A systematic review of currently available evidence. Crit. Rev. Oncol./Hematol..

[9] Michaud D.S., Liu Y., Meyer M., Giovannucci E., Joshipura K. (2008). Periodontal disease, tooth loss, and cancer risk in male health professionals: A prospective cohort study. Lancet Oncol..

[10] Huang H.Y., Lin P.Y., Chen C.C., Chen Y.K. (2024). Risk factors for the development of oral precancerous lesions in a cohort of 293 oral lichen planus patients with or without chronic periodontitis in southern Taiwan. J. Dent. Sci..

[11] Zhou F., Wang Z., Zhang G., Wu Y., Xiong Y. (2024). Immunosenescence and inflammaging: Conspiracies against alveolar bone turnover. Oral Dis..

[12] Ma K.S.-K., Chiang C.H., Chen S.T., Dinh Y., Chiang C.H., Van Dyke T.E., Sullivan R., Ananthakrishnan A.N., Hsia Y.P., Peng C.M. (2024). Periodontitis is an immune-related adverse event associated with immune checkpoint inhibitors: A multi-center cohort study. Cancer Lett..

[13] Huang J., Yang K., Gao L., He Q., Ge S. (2025). Microbial community composition in subgingival plaques and heterogeneity of tumor tissue TCRβ CDR3 repertoire in patients with moderate-to-severe periodontitis and oral squamous cell carcinoma. Technol. Health Care.

[14] Fülöp T., Larbi A., Kotb R., de Angelis F., Pawelec G. (2011). Aging, immunity, and cancer. Discov. Med..

[15] Liu Q., Li J., Sun X., Lin J., Yu Z., Xiao Y., Li D., Sun B., Bao H., Liu Y. (2025). Immunosenescence and cancer: Molecular hallmarks, tumor microenvironment remodeling, and age-specific immunotherapy challenges. J. Hematol. Oncol..

[16] Chen A.C.Y., Jaiswal S., Martinez D., Yerinde C., Ji K., Miranda V., Fung M.E., Weiss S.A., Zschummel M., Taguchi K. (2024). The aged tumor microenvironment limits T cell control of cancer. Nat. Immunol..

[17] He Y., Qiu Y., Yang X., Lu G., Zhao S.S. (2025). Remodeling of tumor microenvironment by cellular senescence and immunosenescence in cervical cancer. Semin. Cancer Biol..

[18] Hayflick L., Moorhead P.S. (1961). The serial cultivation of human diploid cell strains. Exp. Cell Res..

[19] Liu N., Wu J., Deng E., Zhong J., Wei B., Cai T., Xie Z., Duan X., Fu S., Osei-Hwedieh D.O. (2025). Immunotherapy and senolytics in head and neck squamous cell carcinoma: Phase 2 trial results. Nat. Med..

[20] González-Osuna L., Sierra-Cristancho A., Cafferata E.A., Melgar-Rodríguez S., Rojas C., Carvajal P., Cortez C., Vernal R. (2022). Senescent CD4^+^CD28^−^ T lymphocytes as a potential driver of Th17/Treg imbalance and alveolar bone resorption during periodontitis. Int. J. Mol. Sci..

[21] Coppé J.P., Desprez P.Y., Krtolica A., Campisi J. (2010). The senescence-associated secretory phenotype: The dark side of tumor suppression. Annu. Rev. Pathol..

[22] Tchkonia T., Zhu Y., van Deursen J., Campisi J., Kirkland J.L. (2013). Cellular senescence and the senescent secretory phenotype: Therapeutic opportunities. J. Clin. Investig..

[23] Ostrowska K., Niewinski P., Piotrowski I., Ostapowicz J., Koczot S., Suchorska W.M., Golusiński P., Masternak M.M., Golusiński W. (2024). Senescence in head and neck squamous cell carcinoma: Relationship between senescence-associated secretory phenotype (SASP) mRNA expression level and clinicopathological features. Clin. Transl. Oncol..

[24] Ortiz-Montero P., Londoño-Vallejo A., Vernot J.P. (2017). Senescence-associated IL-6 and IL-8 cytokines induce a self- and cross-reinforced senescence/inflammatory milieu strengthening tumorigenic capabilities. Cell Commun. Signal..

[25] Chen S., Zhou D., Liu O., Li Y., Wang H., Zhang G. (2022). Cellular senescence and periodontitis: Mechanisms and therapeutics. Biology.

[26] Li J., Sanui T., Shida M., Yotsumoto K., Ahmad M., Wang Z., Xiao M., Hayashi C., Shinjo T., Taketomi T. (2026). Ligature-induced periodontitis in mice potentially accelerates CD4^+^ T-cell senescence and exacerbates rheumatoid arthritis. Front. Immunol..

[27] Chu X., Elashiry M., Carroll A., Cornelius Timothius C.J., Cutler C.W., Elsayed R. (2025). The role of senescence in experimental periodontitis at the causal level: An in vivo study. Cells.

[28] Aquino-Martinez R., Eckhardt B.A., Rowsey J.L., Fraser D.G., Khosla S., Farr J.N., Monroe D.G. (2021). Senescent cells exacerbate chronic inflammation and contribute to periodontal disease progression in old mice. J. Periodontol..

[29] Wang S., Nie F., Yin Q., Tian H., Gong P., Ju J., Liu J., Yang P., Yang C. (2024). Periodontitis promotes tumor growth and immune evasion via PD-1/PD-L1. Cancer Immunol. Immunother..

[30] Zheng Y., Wang Z., Weng Y., Sitosari H., He Y., Zhang X., Shiotsu N., Fukuhara Y., Ikegame M., Okamura H. (2025). Gingipain regulates isoform switches of PD-L1 in macrophages infected with *Porphyromonas gingivalis*. Sci. Rep..

[31] Groeger S., Denter F., Lochnit G., Schmitz M.L., Meyle J. (2020). *Porphyromonas gingivalis* cell wall components induce PD-L1 expression on human oral carcinoma cells by a RIP2-dependent mechanism. Infect. Immun..

[32] Muñoz-Grez C.P., Vidal M.A., Rojas T.B., Ferrada L.E., Zuñiga F.A., Vera A.A., Sanhueza S.A., Quiroga R.A., Cabrera C.D., Antilef B.E. (2025). Host-microbe computational proteomic landscape in oral cancer revealed key functional and metabolic pathways between *Fusobacterium nucleatum* and cancer progression. Int. J. Oral Sci..

[33] Cao Q., Yang Y., Bai S., Shen J. (2026). The oral bacterial microbiota change in oral squamous cell carcinoma: A systematic review and meta-analysis. BMC Oral Health.

[34] Hanahan D., Weinberg R.A. (2011). Hallmarks of cancer: The next generation. Cell.

[35] Irfan M., Delgado R.Z.R., Frias-Lopez J. (2020). The oral microbiome and cancer. Front. Immunol..

[36] Chen Q., Pang X., Liu K., Wang H., Qiu T., Luo G.F., Shang Z. (2025). *Porphyromonas gingivalis* outer membrane vesicles promote oral tumorigenesis through suppressing innate immune surveillance. Microbiol. Res..

[37] Schwechheimer C., Kuehn M.J. (2015). Outer-membrane vesicles from Gram-negative bacteria: Biogenesis and functions. Nat. Rev. Microbiol..

[38] Toyofuku M., Schild S., Kaparakis-Liaskos M., Eberl L. (2023). Composition and functions of bacterial membrane vesicles. Nat. Rev. Microbiol..

[39] Sun L., Wu J., Du F., Chen X., Chen Z.J. (2013). Cyclic GMP-AMP synthase is a cytosolic DNA sensor that activates the type I interferon pathway. Science.

[40] Ergun S.L., Fernandez D., Weiss T.M., Li L. (2019). STING polymer structure reveals mechanisms for activation, hyperactivation, and inhibition. Cell.

[41] Lai P., Liu L., Bancaro N., Troiani M., Calì B., Li Y., Chen J., Singh P.K., Arzola R.A., Attanasio G. (2025). Mitochondrial DNA released by senescent tumor cells enhances PMN-MDSC-driven immunosuppression through the cGAS-STING pathway. Immunity.

[42] Niklander S.E., Aránguiz P., Faunes F., Martinez B., Esguep A. (2023). Aging and oral squamous cell carcinoma development: The role of cellular senescence. Front. Oral Health.

[43] Demaria M., O’Leary M.N., Chang J., Shao L., Liu S., Alimirah F., Koenig K., Le C., Mitin N., Deal A.M. (2017). Cellular senescence promotes adverse effects of chemotherapy and cancer relapse. Cancer Discov..

[44] Majewska J., Agrawal A., Mayo A., Roitman L., Chatterjee R., Sekeresova Kralova J., Landsberger T., Katzenelenbogen Y., Meir-Salame T., Hagai E. (2024). p16-dependent increase of PD-L1 stability regulates immunosurveillance of senescent cells. Nat. Cell Biol..

[45] Wang T.W., Johmura Y., Suzuki N., Omori S., Migita T., Yamaguchi K., Hatakeyama S., Yamazaki S., Shimizu E., Imoto S. (2022). Blocking PD-L1–PD-1 improves senescence surveillance and ageing phenotypes. Nature.

[46] Xie W., Shen J., Wang D., Guo J., Li Q., Wen S., Dai W., Wen L., Lu H., Fang J. (2022). Dynamic changes of exhaustion features in T cells during oral carcinogenesis. Cell Prolif..

[47] Wang J., Xie T., Wang B., William W.N., Heymach J.V., El-Naggar A.K., Myers J.N., Caulin C. (2017). PD-1 blockade prevents the development and progression of carcinogen-induced oral premalignant lesions. Cancer Prev. Res..

[48] Chen Y., Li Q., Li X., Ma D., Fang J., Luo L., Liu X., Wang X., Lui V.W.Y., Xia J. (2017). Blockade of PD-1 effectively inhibits in vivo malignant transformation of oral mucosa. Oncoimmunology.

[49] You L., Wu Q. (2025). Cellular senescence in tumor immune escape: Mechanisms, implications, and therapeutic potential. Crit. Rev. Oncol./Hematol..

[50] Shi H., Xiao M., Li Y., Liu X., Na J., Liang C., Hua J., Meng Q., Wei M., Wang W. (2025). The role of senescence, its therapeutic relevance and clinical implications in the tumor microenvironment. Theranostics.

[51] Neuzillet C., Marchais M., Vacher S., Hilmi M., Schnitzler A., Meseure D., Leclere R., Lecerf C., Dubot C., Jeannot E. (2021). Prognostic value of intratumoral *Fusobacterium nucleatum* and association with immune-related gene expression in oral squamous cell carcinoma patients. Sci. Rep..

[52] Song X., Wang J., Gu Z., Qiu X., Yuan M., Ke H., Deng R. (2025). *Porphyromonas gingivalis* and *Fusobacterium nucleatum* synergistically strengthen the effect of promoting oral squamous cell carcinoma progression. Infect. Agent Cancer.

[53] Ofuchi T., Hu Q., Omachi K., Kanemitsu K., Otsu H., Yonemura Y., Tobo T., Baba Y., Iwatsuki M., Mimori K. (2026). Intratumoral *Fusobacterium nucleatum* drives cancer-associated fibroblasts enrichment and immune exclusion in esophageal squamous cell carcinoma. Ann. Gastroenterol. Surg..

[54] Childs B.G., Gluscevic M., Baker D.J., Laberge R.M., Marquess D., Dananberg J., van Deursen J.M. (2017). Senescent cells: An emerging target for diseases of ageing. Nat. Rev. Drug Discov..

[55] Xu M., Pirtskhalava T., Farr J.N., Weigand B.M., Palmer A.K., Weivoda M.M., Inman C.L., Ogrodnik M.B., Hachfeld C.M., Fraser D.G. (2018). Senolytics improve physical function and increase lifespan in old age. Nat. Med..

[56] Chander A., Iacovacci J., Pellon A., Kataria R., Grigoriadis A., Maher J., Sears C., Bachrach G., Urbano T.G., Lei M. (2024). *Fusobacterium* is toxic for head and neck squamous cell carcinoma and its presence may determine a better prognosis. Cancer Commun..

[57] Ferris R.L., Spanos W.C., Leidner R., Gonçalves A., Martens U.M., Kyi C., Sharfman W., Chung C.H., Devriese L.A., Gauthier H. (2021). Neoadjuvant nivolumab for patients with resectable HPV-positive and HPV-negative squamous cell carcinomas of the head and neck in the CheckMate 358 trial. J. Immunother. Cancer.

[58] Burtness B., Harrington K.J., Greil R., Soulières D., Tahara M., de Castro G., Psyrri A., Basté N., Neupane P., Bratland Å. (2019). Pembrolizumab alone or with chemotherapy versus cetuximab with chemotherapy for recurrent or metastatic squamous cell carcinoma of the head and neck (KEYNOTE-048): A randomised, open-label, phase 3 study. Lancet.

[59] Cramer J.D., Burtness B., Ferris R.L. (2019). Immunotherapy for head and neck cancer: Recent advances and future directions. Oral Oncol..

[60] Yang Z., Peng Y., Zang J., Wang J., Wang Z., Zhang G., Sun J., Chen H., Xie Y., Peng X. (2025). Senescence-primed ferroptosis enabled by a metal-organic framework nanoplatform for enhanced cancer therapy. ACS Nano.

[61] D’Aiuto F., Orlandi M., Gunsolley J.C. (2013). Evidence that periodontal treatment improves biomarkers and CVD outcomes. J. Periodontol..

[62] Radafshar G., Shad B., Ariamajd E., Geranmayeh S. (2010). Effect of intensive non-surgical treatment on the level of serum inflammatory markers in advanced periodontitis. J. Dent..

[63] Zhang W., Song S., Zhang Y., Pan Y., Hu D., Wang Y. (2026). Emerging strategies in senotherapeutics: From broad-spectrum senolysis to precision reprogramming. npj Aging.

